# Altered processing of self-produced sensations in psychosis at cortical and spinal levels

**DOI:** 10.1038/s41380-025-03130-w

**Published:** 2025-07-25

**Authors:** Paula C. Salamone, Adam Enmalm, Reinoud Kaldewaij, Marie Åman, Charlotte Medley, Michal Pietrzak, Håkan Olausson, Andrea Johansson Capusan, Rebecca Boehme

**Affiliations:** 1 Center for Social and Affective Neuroscience, Department of Biomedical and Clinical Sciences, Linköping University, 58185 Linköping, Sweden; 2 Center for Medical Imaging and Visualization, Linköping University, 58185 Linköping, Sweden; 3 Department for Psychiatry, Linköping University Hospital, 58185 Linköping, Sweden; 4 Department of Clinical Neurophysiology, Linköping University Hospital, 58185 Linköping, Sweden

**Keywords:** Schizophrenia, Neuroscience

## Abstract

Psychosis is often characterized by disturbances in the sense of self, with patients frequently misattributing self-produced sensations to external sources. While somatic hallucinations and misperceptions are common, the underlying disruptions in basic bodily self-processing remain unclear. We aimed to investigate processing of self-evoked sensations, including touch and interoception, in psychosis using a multimodal, multi-method approach. This case-control-study included a total of 70 participants (35 patients diagnosed with psychotic disorders, 35 age- and sex-matched controls). Participants performed self-/other-touch-tasks and interoceptive assessments during functional MRI, evoked potentials measurements, and/or behavioral and psychophysical tests. Primary outcomes included neural and behavioral responses to self- and externally-generated sensations (touch and heartbeat). Brain activation, spinal evoked responses, heartbeat perception and processing (evoked responses), and behavioral measures were analyzed, with preregistered hypotheses. Patients demonstrated heightened right superior temporal gyrus activation during self-touch. Tactile self-other distinction impairments were evident at the spinal cord level. Behaviorally, patients showed reduced differentiation in tactile thresholds for self- vs. other-touch. Interoceptive impairments included diminished cortical responses to heartbeat signals, lower interoceptive accuracy (heartbeat detection), and reduced self-reported interoceptive sensitivity. These findings reveal pervasive sensory and self-related disturbances in psychotic disorders. Impairments in differentiating self- and externally-evoked responses, detectable as early as the spinal cord level, may contribute to higher-order symptoms of psychosis.

## Introduction

Schizophrenia is often described as a disorder of the self [1, 2], involving heightened self-referential thinking [3] and misidentifying self-produced sensations, such as perceiving one’s voice as alien [4]. The self is a hierarchical construct, with the bodily self forming its foundation [5, 6]. A coherent bodily self-experience requires perceiving and identifying signals - proprioceptive, interoceptive, or somatosensory - as one’s own. Somatosensory signals are crucial for distinguishing self-evoked from non-self-evoked sensations, as self-touch and touch from others create the same peripheral stimulus [7]. This distinction is essential for self-functioning and interaction with the world. *Perception and understanding of self* is suggested as a key symptom domain (within “Systems for Social Processes”) in the research domain criteria (RDoC) [8].

We asked whether self-related symptoms in psychosis stem from a dysfunction in distinguishing self- from non-self-generated sensations. Using a multimethod approach, we assessed neural and behavioral responses to self- and non-self-generated sensations, exploring whether patients show alterations in self-related processes, at which level these occur, and their relation to symptomatology. Following the RDoC recommendations, we aimed for a more dimensional approach and included patients spanning the different psychotic disorders within the F-20 diagnoses.

Previous studies have reported sensory domain alterations related to the bodily self in schizophrenia, yet these findings often lack integration across sensory modalities, and their neural underpinnings remain unclear. For instance, interoceptive impairments [9–11], have been linked to positive symptoms [10, 12], though findings are inconsistent [11] and may be confounded by cognitive factors. This can be addressed by a different task design paired with physiological measures like the heartbeat evoked potential (HEP). While altered HEPs in schizophrenia were reported, no behavioral measures were included [13].

Tactile stimulation activates the somatosensory cortex, and social-affective touch also engages the insula [14]. Self-touch is processed differently [15], likely due to its high predictability, leading to attenuated perception [16]. This attenuation is diminished in schizophrenia [17, 18], which might relate to positive symptoms [19] and correlates with altered neural processing [20]. However, previous studies used tool-mediated setups, which, while controlled, lack ecological validity. Skin-to-skin touch offers a closed-loop for investigating sensory attenuation with greater relevance to real-world interactions.

The prevailing model of self-touch attenuation states that sensory outcomes are predicted, and when predictions match sensory outcomes, perception is attenuated [19]. We found differences between self- and non-self-touch already at the spinal cord level [15] possibly due to modulation by top-down processes like predictions [21]. Sensory attenuation might be a factor mediating self-other-distinction: self-evoked sensations are always more predictable than externally evoked sensations. In line with this, the degree of neural self-other-touch distinction relates to higher order self-concept [15], and the dissociative substance ketamine decreases this neural self-other-difference [22].

Differences in sensory attenuation may reflect impaired predictive modeling in schizophrenia, for instance, it might result from weak low-level priors, causing imprecise predictions and prediction errors. This might be compensated for by strong higher order priors [23], which then form the basis of delusional beliefs that explain away prediction errors [24].

Here, we evaluated sensory distinctions between self- and externally generated sensations in touch and interoception. We used an ecological task with self-touch and other-touch during brain imaging, and during neurophysiology (evoked potentials) of brain and spinal cord. We measured sensory thresholds during self-touch and other-touch. We assessed interoception versus exteroception through behavior, electrophysiology, and self-report. The measures regarding interoception can be understood as mirroring the touch measures: they compare a self-generated (heartbeat) versus a non-self-generated (heartbeat-tone) stimulus. Our preregistered hypothesis was to find altered distinction of self- and non-self-generated sensations (https://osf.io/kzscj/). For brain imaging analysis, we used both a whole-brain approach and pre-registered anatomical regions of interest known to be involved in touch processing, self-other-distinction, and bodily self processing (right posterior superior temporal gyrus (STG) [25], insula [26], anterior cingulate cortex [27], primary somatosensory cortex [14], and precuneus [28]). In an exploratory analysis, we also included a functionally defined ROI of a temporoparietal cortex (TPC) region that showed significant effects in our previous pharmacological model of an altered self using the same task [22].

## Methods

### Procedure

**Fig. 1:**
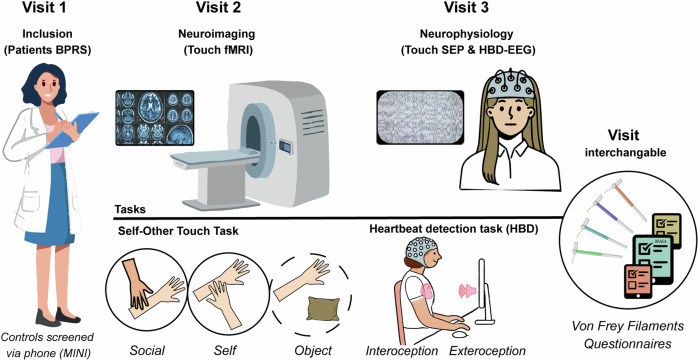
Procedure. Patients came for three visits, controls for two. The initial visit for patients included an interview using the Brief Psychiatric Rating scale (BPRS), while controls were screened over the phone using MINI-interview. fMRI functional magnetic resonance imaging, SEP somatosensory evoked potentials, HBD heartbeat detection task, EEG electroencephalogram.

An overview of the procedures is depicted in Fig. 1. More details on all methods are provided in the supplement. If not stated otherwise, analyses were preregistered (https://osf.io/kzscj). For the brain imaging analysis, we pre-registered five regions of interest (ROIs) based on their known involvement in somatosensory and self-related processing (right posterior STS [25], insula [26], anterior cingulate cortex [27], primary somatosensory cortex [14], precuneus [28]).

Appropriate parametric analyses were done unless equal variance was violated, in case the appropriate non-parametric tests were conducted, and reported accordingly. Effect sizes for all comparisons are reported using the appropriate statistical metric. For Cohen’s D and rank biserial correlations (r_rb_), effect sizes can be interpreted as small at 0.2, medium at 0.5, and large at 0.8. For partial eta squared (η²), 0.01 is considered a small effect, 0.06 a medium effect, and 0.14 a large effect. For Cramer’s V, effect sizes can instead be considered small below 0.2, medium between 0.2 and 0.6, and large at 0.6 and above.

### Ethics approval and consent to participate

The study was approved by the responsible Swedish Ethics Authorities (regionala etikprövningsnämnden i Linköping and Etikprövningsmyndigheten, Dnr 2019-00207, amendments 2020-05535, 2021-00572, 2021-06581-02, 2023-05270-02). Methods were performed in accordance with the relevant guidelines and regulations. Before initiation of study procedures, written informed consent was obtained from all participants.

### Participants

A sample size of 35 patients and 35 controls was considered appropriate based on our previous studies employing the same tasks in other psychiatric populations [15, 29–32] and taking into account potential drop out of individuals during fMRI.

### Patients

Recruited from outpatient psychiatric clinics in Östergötland, Sweden. Inclusion criteria: psychotic disorder (DSM-IV, F20-F29), age 18–50. Exclusion criteria: substance use (current/past 6 months), insufficient Swedish skills, intellectual disability, medical issues affecting left arm sensations.

### Controls

Recruited via social media, flyers, and a participant database. Inclusion criteria: no psychiatric disorders or major health issues, age 18–50. Exclusion criteria: same as above.

### fMRI

The touch paradigm consisted of three conditions, each presented 10 times in randomized order and lasting 12 s per trial: self-touch, other-touch, and object-touch for movement control. Participants performed or received slow, gentle strokes on their left forearm, or touched a pillow respectively. The experimenter was always a woman, standing next to the scanner where she received auditory cues on when to perform the other-touch condition. Participants were cued on when to perform the touch by text visible on screen through MR-compatible goggles (VisuaStim Digital; Resonance Technologies). The protocol for image acquisition, scanner settings, and preprocessing was identical to our previous studies employing this task [15, 22, 29, 31].

The first level model included the cue-phase, the touch-phase, a regressor for touch offset one second after each active condition, and realignment parameters. In addition, the first temporal derivative of realignment parameters in x,y,z-directions and a regressor censoring volumes with *>*1 mm volume-to-volume movement were added to the model, to account for the potential of increased movement.

To compare self-other-distinction between groups, we calculated a contrast between self-touch (motion corrected by using self>object-touch contrast) and other-touch at the first level. At the second level a t-test compared groups. To assess the differences between groups independent of condition, a flexible factorial ANOVA with the factors group and condition (self, other, object) was used. To compare the conditions separately, between-group t-test was used for the other-touch condition and the self-touch condition separately.

Results were corrected for multiple comparisons using the family-wise error (FWE) correction at whole brain level. In addition, we performed small volume FWE-correction (SVC) for a-priori pre-registered regions of interest (ROI, anatomically defined): right posterior superior temporal gyrus (STG), insula, anterior cingulate cortex, primary somatosensory cortex, and precuneus. In an exploratory analysis, we included a functionally defined ROI of a temporoparietal cortex (TPC) region that showed significant effects in our previous pharmacological model of an altered self using ketamine and the same task [22].

### Somatosensory Evoked Potentials (SEP)

As established previously [15, 30], SEPs were recorded during four conditions: baseline, self-touch, other-touch, and object-touch. Electrical stimulation targeted the radial nerve at the base of the left thumb, with 300 pulses delivered during two runs per condition. During baseline, only the electrical pulses occurred. During the touch-conditions, participants performed with the right hand (self- and object-touch) or received touch from the experimenter (other-touch), like in the fMRI experiment. Touch occurred on the left arm targeting the same dermatome as the electrical pulses, i.e. innervated by the radial nerve. Touch continued during a run of ca. 1 min.

Responses were recorded for 100 ms after each pulse. Reponses were averaged across pulses and runs per condition. Analysis focused on amplitude and latency of the N13 (cervical) and N20 (cortical) components. The touch-conditions were corrected for baseline to account for differences in height and nerve conductance velocity. A repeated-measures ANOVA with the factors condition and group was used. An exploratory between-group Mann-Whitney-test on self-other difference was calculated.

### Touch thresholds

As established previously [15, 22], touch thresholds were assessed using von Frey monofilaments (BIO-VF-M, Bioseb, Force of 0008–10 g/0.078–98.066 mN) across) on the left dorsal forearm during four conditions: baseline (no touch), self-touch, other-touch, and object-touch. Participants sat relaxed with eyes closed and indicated verbally when they perceived the filament stimulation to their left arm. At baseline, only stimulation with the filament occurred. During the touch conditions, participants touched their own left arm (self-touch) or were touched by the experimenter (other-touch) on the left arm, simultaneously with the filament stimulation that occurred in random locations in the same area of the arm that was being touched. During object-touch, the participant touched a pillow with their right hand, while stimulation with the filament occurred on the left arm. Thresholds were determined by the smallest filament detected in ≥5/10 applications.

A 2 × 2 ANOVA with factors group and condition was performed. An exploratory between-group Mann-Whitney-test on self-other difference was calculated.

### Interoception

#### Heartbeat detection

Electrocardiogram (ECG) was recorded. In the interoceptive condition, participants tapped a button upon perceiving their own heartbeat, with no external clue. The exteroceptive condition served as a control, where participants tapped in sync with a pre-recorded heartbeat sound (60bpm, irregular intervals). Each block lasted 2 min, with condition order counterbalanced. Accuracy was measured by tapping-cardiac synchronization (see supplement for accuracy and confidence score calculations) [32–34]. A 2 × 2 ANOVA was conducted, followed by post-hoc Tukey tests.

#### Heartbeat-Evoked Potentials (HEP)

EEG and ECG signals were recorded. Participants watched a monitor with a plus-sign. They were instructed to listen to a pre-recorded heartbeat (exteroception, as above), or to focus on feeling their own heartbeat (interoception) for 2 min. The order was counterbalanced.

HEP was evaluated through combined EEG-ECG-analysis. We compared overall HEP modulation across conditions (interoception versus exteroception) and between groups using a point-by-point Monte Carlo permutation test with bootstrapping [35].

#### Combined analyses

Combined analyses were conducted to explore the relationship between experimental measures and symptomatology (not pre-reregistered). Linear regressions predicted BPRS scores using group-differentiating experimental measures, i.e. extracted beta values from STG during self-touch and from TPC during other-touch, self-other-difference SEP at the cervical spinal level and during threshold estimation, HEP area under the curve during interoception, and HBD accuracy during interoception. Exploratory analyses on the BPRS subscales (positive, negative, affective) were performed and results are presented in the supplement. Logistic regression assessed whether the neural measures predicted group membership.

#### Control analyses

Extensive control analyses to check for medication and diagnosis effects were performed. Details on these and their results are presented in the supplement. In short, to check for medication effects during fMRI, chlorpromazine equivalents were correlated with blood oxygen level dependent (BOLD) signal during each condition. For all measures that differed between groups linear regressions were performed onto these measures to check for effects of medication (chlorpromazine equivalents) and diagnosis. To control for antipsychotic medication classes, the main analyses were repeated without the two patients receiving first generation antipsychotics. Furthermore, the groups receiving second and third generation antipsychotics were compared on the measures that differed between groups. In addition, the two subgroups that received / did not receive antidepressants were compared on these measures and fluoxetine equivalents were correlated with these measures. Finally, to control for illness duration, years since diagnosis were regressed onto the measures that differed between groups.

## Results

### Demographics and symptoms

60 patients expressed interest. 4 could not be reached, 11 withdrew interest after study description. Forty-five patients were scheduled for inclusion, 7 withdrew participation prior to inclusion, one did not meet inclusion criteria, and one withdrew after inclusion but prior to experimental procedures. 35 patients enrolled in the study (figure S1).

Diagnoses included schizophrenia (n = 14), schizoaffective disorder (n = 9), non-specific non-organic psychosis (n = 8), delusional disorder (n = 3), and acute schizophrenia-like psychosis (n = 1). Control analyses did not reveal differences between diagnosis groups, except for the HEP AUC (see supplement). All patients were on antipsychotic medication (mean chlorpromazine equivalent 371.2 ± 216.5 mg, first generation n = 2, second generation n = 19, third generation n = 12, second and third generation n = 2. 19 patients were on stable antidepressants (see supplement). Patients had a mean illness duration of 4.8 ± 4.5 years and mean BPRS score of 33.1 ± 7.3.

85 controls expressed interest. 55 were eligible for participation. The 35 best matching for sex and age were included (figure S1).

Group differences are summarized in Table 1. The psychosis group showed significant differences in the interoceptive awareness questionnaire (total score and non-worrying subscore), indicating lower interoceptive awareness. They showed more touch aversion (STQ) and lower registration on the sensory profile, indicating that patients experienced more overstimulation and difficulties in a noisy environment.

**Table 1:** Participant demographics and self-report outcomes.

Basic Demographics	Controls	Patients	Test outcome	Significance	Effect size
Gender	15 F/20 M	15 F/20 M	χ^2^ (1) = 0.0	p = 1.000	V = 0.000
Age	35.7 (9.3)	35.5 (7.4)	U = 624.0	p = 0.897	d = 0.239
BMI	24.3 (4.3)	29.2 (6.1)	t (68) = −3.882	p < 0.001*	d = 0.264
**Handedness**			χ^2^ (2) = 5.581	p = 0.061	V = 0.282
Right	34 (97.1%)	28 (80%)			
Left	0 (0%)	4 (11.4%)			
Ambidextrous	1 (2.9%)	3 (8.6%)			
**Education**			χ^2^ (2) = 7.921	p = 0.019*	V = 0.336
Primary	0 (0%)	6 (17.1%)			
Secondary	13 (37.1%)	15 (42.9%)			
Tertiary	22 (62.9%)	14 (40%)			
**Occupation**			χ^2^ (2) = 22.2	p < 0.001*	V = 0.588
Unemployed / sick	0 (0%)	18 (51.4%)			
Student	10 (28.6%)	5 (14.3%)			
Working	25 (71.4%)	12 (34.3%)			
**Questionnaires**					
MAIA	101.5 (21.5)	87.4 (18.3)	t (68) = 2.952	p = 0.004*	d = 0.706
MAIA: Noticing	3.6 (0.8)	3.2 (0.9)	t (68) = 1.964	p = 0.054	d = 0.469
Maia: Non-distracting	2.4 (0.8)	2.1 (0.8)	t (68) = 1.787	p = 0.078	d = 0.427
MAIA: Non-worrying	3.1 (0.9)	2.3 (1.2)	t (68) = 2.941	p = 0.004*	d = 0.703
MAIA: Attention regulation	3.2 (0.9)	2.9 (0.9)	t (68) = 1.243	p = 0.218	d = 0.297
MAIA: Emotional awareness	3.4 (0.9)	2.9 (1.1)	t (68) = 2.024	p = 0.047	d = 0.484
MAIA: Self-regulation	3.0 (1.0)	2.5 (1.1)	t (68) = 1.927	p = 0.058	d = 0.461
MAIA: Bodily listening	2.5 (1.1)	2.5 (1.2)	t (68) = 0.244	p = 0.808	d = 0.058
MAIA: Trusting	3.8 (1.0)	3.0 (1.4)	U = 823.5	p = 0.013	r_rb_ = 0.344
STQ	37.2 (9.1)	44.1 (14.0)	U = 442.5	p = 0.046*	r_rb_ = −0.278
EQ	46.1 (13.0)	41.4 (12.8)	t (68) = 1.510	p = 0.136	d = 0.361
SP: Low Registration	28.5 (5.6)	33.9 (7.5)	U = 335.0	p = 0.001*	r_rb_ = −0.453
SP: Sensation Seeking	42.6 (5.9)	41.1 (7.5)	t (68) = 0.905	p = 0.369	d = 0.216
SP: Sensation Sensitivity	33.1 (5.6)	37.9 (9.5)	U = 406.5	p = 0.016	r_rb_ = −0.336
SP: Sensation Avoiding	36.1 (7.1)	40.7 (9.1)	t (68) = −2.401	p = 0.019	d = −0.574
SP: Touch processing	28.6 (4.1)	30.4 (6.6)	U = 522.5	p = 0.292	r_rb_ = −0.147

Demographics and questionnaire results, compared between groups. Test outcome denotes which analysis was done for each variable. The Multidimensional assessment of interoceptive awareness (MAIA) subscales and Sensory profile (SP) subscales were corrected for multiple comparisons using Bonferroni-Holm correction.

*STQ* social touch questionnaire, *EQ* emotional quotient.

*Significant difference.

### Increased neural responses to touch in patients

**Fig. 2:**
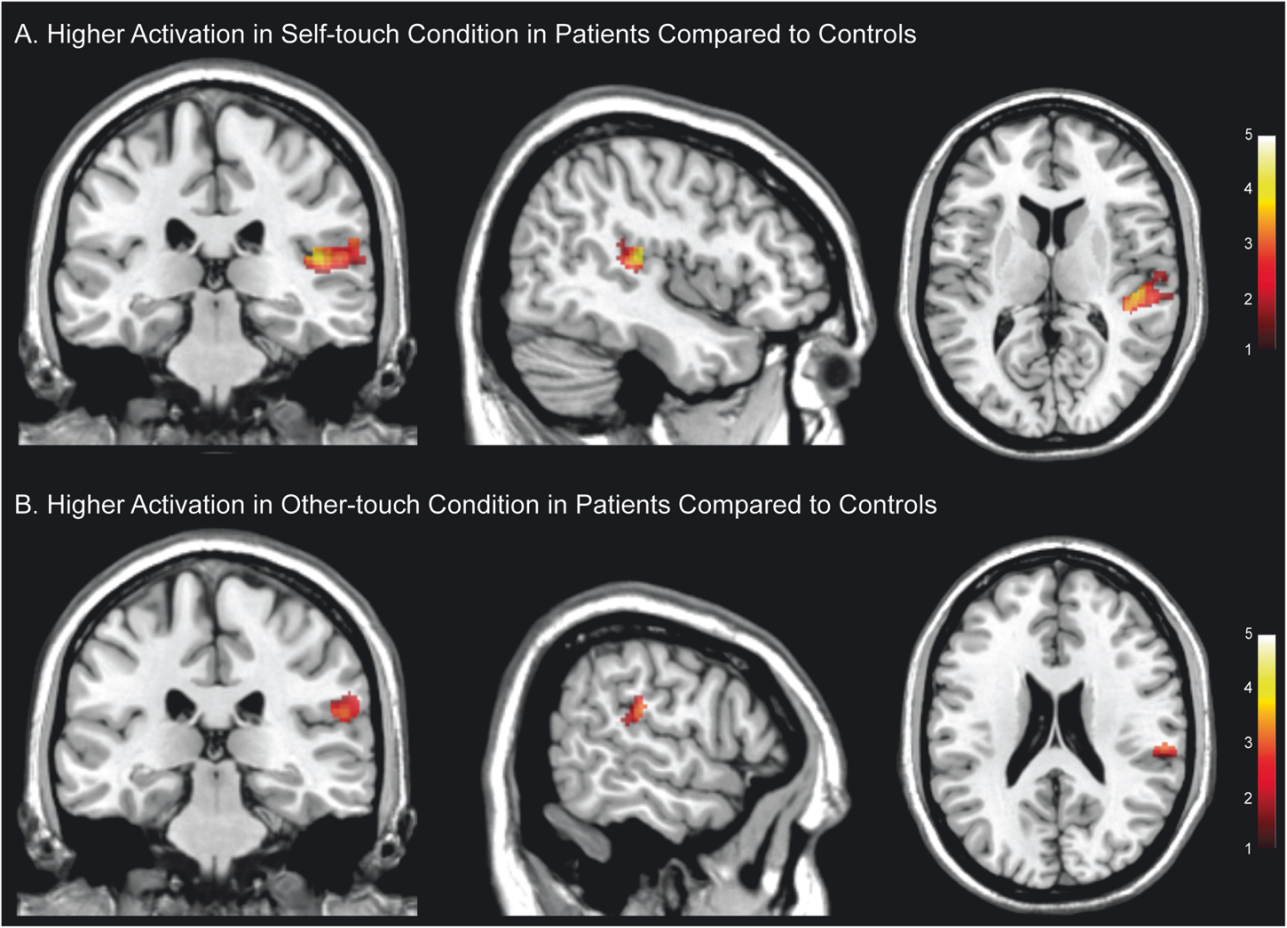
Brain activity differences. The patient group compared to the controls showed increased activity increased during (**A**) self-touch in the superior temporal gyrus (MNI_xyz_ = 45, −28, 11) and during (**B**) other-touch in the temporo-parietal cortex (MNI_xyz _= 59, −28, 22). Two sample t-test between groups, thresholded at p < 0.001 for display purpose, color-bar indicates t-values.

Patients showed higher activation in response to touch compared to controls, primarily in sensory and associative brain regions, including the occipital cortex, middle temporal gyrus, fusiform gyrus, postcentral gyrus, precentral gyrus, supramarginal gyrus, and superior parietal lobe (main effect of group over all conditions at the whole brain level, *p*_FWE_ < 0.05, Table S5, Figure S2). Controls exhibited greater activation in the right occipital cortex, right calcarine cortex, and right cuneus compared to patients (Table S5). The pre-registered analysis of the BOLD signal during self-touch revealed that patients exhibited significantly higher activation in the right STG (*p*_FWE(SVC)_ = 0.028, MNI_xyz_ = 48,−26,14; *p*_FWE(SVC)_ = 0.042, MNI_xyz_ = 42,−30,16, between group t-test, Fig. 2A), potentially reflecting altered predictive coding.

Exploratory analysis of other-touch revealed greater activation in the TPC in patients (p_FWE(SVC)_ = 0.049, MNI_xyz_ = 56, −26,20, between group t-test, Fig. 2B).

The group comparison of the difference between self- and other-conditions did not show significant group effects.

### Reduced latency difference between self- and other-touch at the spinal cord level

**Fig. 3:**
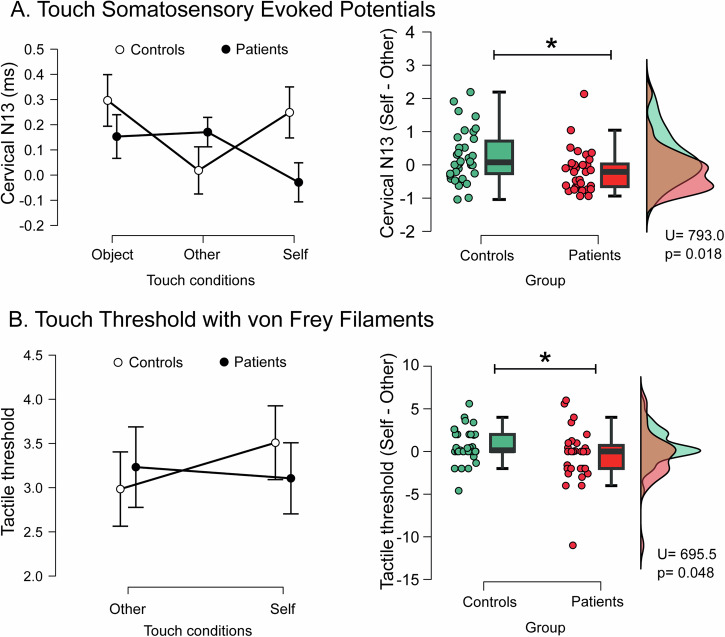
Self-other touch task during somatosensory evoked potentials (SEPs) and touch detection thresholds. **A** Spinal cord SEP corrected for baseline (N13): Significant condition by group interaction in N13 latency (repeated measures ANOVA, left). Self-other difference was lower in patients (between group Mann-Whitney-test, right). **B** Touch detection thresholds: No main effect of condition or group (left). Self-other difference in thresholds was lower in patients compared to controls (between group Mann-Whitney-test, right).

In the spinal cord, there was a significant condition x group interaction (F(2128) = 3.119, p = 0.048, η² = 0.014) on N13 latency. Follow-up group comparison revealed significantly smaller latency differences between self and other in patients (U = 793, p = 0.018, rank-biserial *r* = 0.33, Fig. 3A), suggesting impairments in early sensory processing.

### Reduced difference between detection thresholds during self-other-touch

An exploratory analysis revealed a significant group effect for threshold differences during self- and other-touch (U = 695, p = 0.048, rank-biserial *r* = 0.28, Fig. 3B). Unlike controls, who exhibited higher thresholds during self-touch, patients showed comparable thresholds across conditions, indicating altered sensory attenuation mechanisms.

### Reduced interoceptive accuracy

**Fig. 4:**
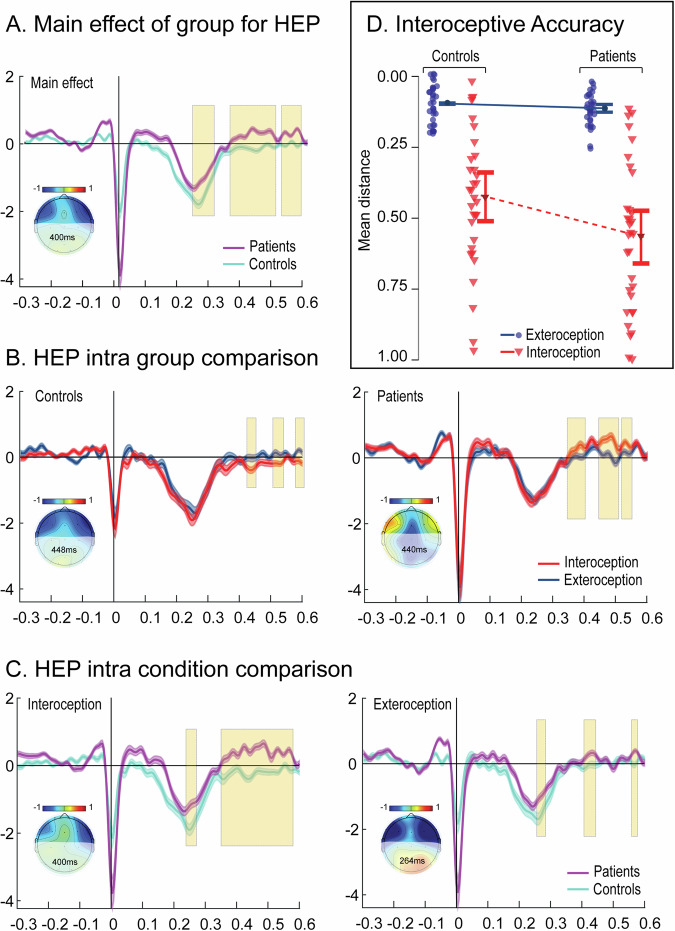
Heartbeat Detection Task, Cortical and Behavioral Measurements. **A** Main effect of group for HEP (Monte Carlo permutation test): Comparing controls (teal) and patients (purple). **B** HEP intra-group comparison: Controls (left) and patients (right). Interoception (red) vs. exteroception (blue). **C** HEP intra-condition comparison: Interoception (left) and exteroception (right). Patients (purple) vs. controls (teal). Mean modulations shown with standard error of the mean (SEM, shadowed lines). Significant time windows marked with yellow shading. Topographical maps highlight activity differences at indicated time points. **D** Interoceptive accuracy: Mean distance indices for patients (red) and controls (blue). Interoception (triangles) vs. exteroception (circles) show significant main effect of group and condition, and interaction effects (repeated measures ANOVA). Controls had higher accuracy than patients in interoception (post-hoc Tukey test). No group differences in exteroception. Both groups had better exteroception accuracy versus interoception. Error bars represent SEM.

Patients exhibited reduced interoceptive accuracy (main effect of group: *F*(1127) = 6.430, p = 0.012, η^2^ = 0.022, group x condition: *F*(1127) = 4.051, p = 0.046, η^2^ = 0.014). A post hoc test confirmed a significant group difference in the interoceptive condition (t = −3180, p = 0.01, Cohen’s *d* = –0.795, Fig. 4D), where patients displayed lower accuracy.

### Altered interoceptive HEP modulation

Patients showed lower HEP amplitudes across three time windows (236–30 ms, 356–500 ms, 520–580 ms, Fig. 4A). Patients did modulate HEP differently for intero- and exteroception, but showed the opposite pattern of controls, i.e. modulation was higher during exteroception than interoception(Fig. 4B).

For interoception, patients showed lower HEP modulation over a longer period (240–272 ms, 352–580 ms) than during exteroception (256–284 ms, 408–444 ms, Fig. 4C).

### Relation to symptomatology and group membership

Touch-related measures significantly predicted the BPRS total scores (F(4,24) = 2.94, p = 0.045, adjusted R^2^ = 0.237), with touch threshold difference between self- and other-touch showing the strongest predictive value (t = −2.14, p = 0.049), while STG activity during self-touch and SEP spinal latency difference were slightly above significance threshold (STG: t = 2.05, p = 0.053; SEP: t = 1.67, p = 0.111). We further explored the relation of the touch-measures with BPRS subscores, and found that they predicted negative symptomatology (see supplement).

Neural measures significantly predicted group membership (X^2^ = 19.37, p < 0.001) with STG activation during self-touch as the strongest predictor (Wald statistic = 7.6, p = 0.006, Odds Ratio 57.96, Table S6). The model explained 42.1% of the variance (Nagelkerke R^2^) and predicted 70.6% of the cases correctly.

#### Control analyses

Detailed results from the control analyses for medication and diagnosis can be found in the supplement. In short, we found no clear effect of medication (antipsychotic and antidepressant) or diagnosis or illness duration on our main findings surviving correction for multiple comparison.

There were a few potential relationships in these exploratory analyses, which did contain very small subsamples: HEP modulation during interoception showed a relation to diagnosis, it was larger in the subgroup with non-specific psychosis (n = 6). Brain activity in the TPC during other-touch was larger in the group receiving second-generation (n = 17) vs. third-generation (n = 11) antipsychotics (when excluding n = 2 on first generation antipsychotics). This might have been driven by lower negative symptoms in this subgroup, since beta-parameter estimates during other-touch in the TPC in turn negatively correlated with the negative symptom subscale (r = −0.406, p = 0.032).

## Discussion

Patients with psychotic disorders displayed reduced neural attenuation of self-evoked sensations, and a smaller difference between self- and non-self-evoked signals already at the spinal cord level. This was accompanied by smaller differences in perceptual thresholds during self- vs. non-self-evoked touch. In the second sensory domain, interoception, patients displayed lower accuracy in detecting the signals originating from self (i.e. heartbeats) and a dysfunctional modulation of the neural markers of these signals (HEP). This was especially pronounced during interoceptive focus. These alterations align with predictive coding models of psychosis, suggesting a shared mechanism underpinning deficits in self-referential processing. Touch-related variables were also associated with symptom severity, and neural measures reliably predicted group membership.

We did not observe the expected smaller self-other distinction in touch-related brain activity. Instead, patients showed increased activity during both touch conditions. Patients exhibited specific alterations: elevated neural activity in the right STG during self-touch and in a right temporoparietal cluster during other-touch. These differences may stem from early-stage or low-level touch processing dysfunction, potentially at the spinal cord level, as suggested by neurophysiological findings of reduced latency differences between self- and other-touch at the cervical spine, driven by faster self-touch latencies.

In interoception, patients showed multidimensional alterations: reduced cortical interoceptive modulation (HEP amplitude), decreased interoceptive accuracy (HBD task), and lower sensitivity (MAIA), aligning with our hypotheses and previous findings.

Our findings of reduced attenuation of self-evoked sensations in psychosis extend previous research [19, 20], demonstrating these deficits across both cortical and spinal levels. Altered spinal processing, reflected by reduced latency differences between self- and other-touch, highlights dysfunction in early-stage sensory processing, potentially originating at the dorsal horn [36]. This novel observation suggests that sensory abnormalities in psychosis may begin at subcortical levels and influence higher-order processes.

Previous studies reported also an overall reduced neural response to touch [20], while we found overall increased response. This difference may stem from their use of mechanical tapping, engaging different pathways than our affective touch stimulus [14]. The nature of touch (social vs. non-social) likely influences patients’ general response but diminished self-touch attenuation appears consistent across touch types.

During self-touch, patients showed increased STG activation, which significantly predicted group membership. In contrast, healthy populations typically show STG deactivation during self-touch [15, 31]. Increased STG activation suggests a mismatch between sensory predictions and incoming signals, potentially driven by weak priors or disrupted top-down modulation. The superior temporal areas are involved in various functions, including speech processing, biological motion [37], and social interaction [38]. STG abnormalities are implicated in hallucinations and self-other distinction [39–41], supporting this region’s role in psychotic symptomatology. A potential common underlying mechanism might be related to predictive processing [42]. These findings have led to suggestions of STG as a target for neurofeedback intervention to reduce self-symptomatology [43]. Therefore, the here reported increased STG response to self-touch in patients may result from a mismatch between prediction and sensation, potentially due to faulty predictions [44], altered bottom-up sensation, or a failure to integrate sensory evidence and update predictions [45].

Altered SEP latencies suggest a breakdown in self-other distinction at the spinal level, potentially driven by disruptions in predictive modulation from cortical circuits. Few studies have examined cortical SEPs in this group, with mixed results [46–50]. Our SEP design might be more sensitive as it integrates evoked potentials with additional touch stimuli and applies baseline correction removing bias by factors like height and nerve conduction speed.

In controls, simultaneous other-touch reduces SEP latency in both the spinal cord and brain compared to self-touch [15]. The posterior cervical spine N13 is thought to reflect the postsynaptic potential of dorsal horn interneurons [51]. Modulations of N13 might be due to changes in dorsal horn excitability [52] depending on the context (i.e. touch from self vs from others). Here, we replicated the self-other-difference effect in controls and found that it was absent in patients, suggesting alterations in the dorsal horn. There are different potential explanations for this lack of modulation: spinal dorsal horn processing could be locally altered, excitability modulation could be altered directly through different top-down signaling or indirectly through alterations of broader physiological processes known to be associated with social touch (among others: increase in parasympathetic activity [53], slowing of heartrate [54]).

Cortical dysfunction or altered predictive processing [55] may influence spinal cordprocessing, as somatosensory circuits in the spinal cord receive significant input from cortical neurons [36]. Similar spinal cord-level changes have been linked to increased somatosensory sensitivity in an autism animal model [56], and at least one gene (GABRB3) implicated in this model is associated with schizophrenia risk [57]. These findings align with previously and here reported generalized sensory abnormalities and higher touch aversiveness in psychosis patients [58].

Studies on audition and vision in schizophrenia report altered evoked potentials, with reduced gating in paired-pulse designs [59, 60], reflecting difficulties attenuating irrelevant stimuli. This aligns with our findings of increased activity and shorter latency during self-touch, a highly predictable and usually irrelevant stimulus for controls.

To test basic tactile sensitivity, we measured detection thresholds and found no baseline differences between patients and controls but a significantly smaller threshold difference between self- and other-touch in patients. This supports altered integration of additional stimuli during self- and other-touch, consistent with our SEP findings and overall results.

Our findings underscore the importance of targeting sensory dysfunction therapeutically, potentially through sensory training or pharmacological modulation of spinal circuits.

Patients exhibited interoceptive processing alterations both behaviorally and neurally, which aligns with a disrupted bodily self-representation in psychosis. Patients showed reduced interoceptive accuracy (i.e. specific to self-generated stimuli), with no impairments in exteroceptive trials, aligning with previous research [10, 11]. The lack of group differences in exteroceptive conditions suggests the results were not due to cognitive or motor impairments.

Reduced HEP modulation further supports this, as HEP reflects cortical processing of interoceptive signals integral to maintaining allostasis and self-awareness. The overall directionality is consistent with resting-state HEP-findings [13]. Monitoring one’s heartbeat is crucial for regulating allostasis, brain activity, responsivity, and the bodily-self model [61, 62]. Reduced HEP modulation, linked to less accurate bodily signal perception, may reflect or contribute to a faulty bodily-self model, as larger HEP modulations relate to self-referential thoughts [63] and are diminished in depersonalization [64]. The reversal of modulation patterns in patients suggests a maladaptive predictive coding mechanism, where interoceptive priors may dominate over sensory evidence. Such dysfunctions could underpin depersonalization and impaired self-referential processing in psychosis.

Taken together, psychosis patients showed altered processing of self-originating stimuli across domains. Following predictive processing theories in schizophrenia [24, 55], the increased neural response to self-touch may result from faulty predictive coding, such as weak low-level priors or failure to update predictions due to strong high-level priors. Aberrant sensations from such dysfunctions may underlie psychotic symptoms, as suggested by the aberrant salience hypothesis [65] and patients’ prodromal experiences [66]. The aberrant salience hypothesis states that schizophrenia patients might experience some perceptions and sensations as salient that should be considered irrelevant [67]. An accumulation of such experiences might lead to the development of delusions, i.e. mental frameworks that explain away the unusual experiences. The aberrant salience attribution might be driven by increased dopaminergic signaling in the mesolimbic system [68–70]. Note, that recent discussions argue that aberrant prediction errors (and in consequence aberrant attentional salience), not incentive salience per se, seem to better explain findings in schizophrenia [71]. Corlett and Fraser write: “…a disconnect between the limbic loop and the associative and sensorimotor loops may result in a lack of causal meaning for actions executed and needing to post-hoc assign meaning to one’s own behavior that apparently occurs without valid meaning” [71].

Sensations evoked by self-touch and the own heartbeat are two types of typical stimuli that should be considered “not relevant” in most occasions. They should also not or rarely evoke prediction errors as they are highly predictable and unsurprising since self-generated [7]. A dysfunctional neural response to these self-generated stimuli is well-aligned with the aberrant salience hypothesis (and also its recent updates). Interestingly, psychosis patients were less accurate in detecting their own heartbeat, indicating that the increased neural response to self-generated sensations does not indicate a *better* perception of internal sensations, but instead a potentially aberrant perception. An explanation could be that for example other internal sensations are misinterpreted and/or mixed up with the heartbeat. This is also in line with the self-reported decreased interoceptive awareness and higher score in the sensory profile subscale “low registration” (i.e. they are more likely to be overstimulated / have a harder time to select information of importance in a noisy environment). Altered priors and altered salience attribution might both affect top-down modulation of spinal cord excitability as it has been shown that expectations can affect spinal cord evoked potentials [21] – a potential explanation for the here observed reduced difference between spinal evoked potentials during self- and other-touch.

Touch-related measures predicted symptomatology, particularly in the negative domain. This differs from prior findings linking reduced attenuation to positive symptoms, possibly due to our medicated, chronic-phase sample with low positive symptoms. Interoceptive self-models likely rely on multiple interoceptive sensations, not heartbeat alone [72]. Touch, bridging extero- and interoception [73], is central to predictive models of self and world. The STG’s increased activation during self-touch and altered spinal cord self-touch processing further highlights the importance of self- and touch-related processes in schizophrenia.

Some limitations need to be considered: Our sample showed low symptomatology, was medicated, and had the condition for several years. Antipsychotic medications work primarily through dopamine D2 receptor antagonism and may influence sensory perception or motor output through alterations in gating and cortical excitability/inhibition [48]. However, previous studies suggest that antipsychotic medication does not cause but normalizes pre-existing disturbances [74–77]. While we cannot exclude that our observed effects were driven by medication, control analyses did not reveal any clear effect of medication dosage or class (supplement). Exclusion of one patient on first generation antipsychotics rendered functional imaging group differences weaker. The overall directionality and location of the group differences remained the same, but was now slightly below significance threshold. However, considering sample size and patient group variance, it is unclear whether this could be due to medication class specific effects. There was also no difference between groups receiving or not receiving antidepressants. In addition, we did not find differences between patients and controls in the object-touch condition and the exteroception condition, which can be understood as control conditions for potential basic sensory and motor alterations. Similarly, these conditions can be understood as a control for potential cognitive deficits. We did not collect IQ or other cognitive measures to control for executive dysfunction, however, the tasks were specifically designed to be simple and easy to follow without a large cognitive load. The touch task was trained beforehand and highly intuitive, and the interoception task used simple button presses instead of heartbeat counting reducing working memory dependence. Since patients did not differ in the exteroceptive signal tracking condition and the object touch condition, it appears safe to presume they were well equipped to perform the tasks.

Self-reported sensory sensitivity and avoidance were higher in the patient group, which might affect social touch processing. We included patients of any psychotic disorders (DSM F20-29), which may introduce variability in findings but enhances generalizability. In the control analyses, we only found a potential difference for one diagnostic sub-group, however, this was the case for a diagnosis that is typically given while patients are still in an earlier phase of being diagnosed (supplement). This might indicate that there is a larger modulation of the HEP in earlier phases of the illness, however, we did not find support for this in an exploratory analysis of time since diagnosis (supplement). The sub-groups of the different diagnoses were very small, reducing interpretability. Similarly, previous studies have found only limited neural differences between schizophrenia and schizoaffective disorders [78].

Psychosis patients display significant dysfunction in the processing and perception of self-evoked sensations, across touch and interoception, evident at cortical and subcortical levels. We suggest a dysfunctional bodily-self predictive model as the shared underlying mechanism. Our findings support predictive coding theories, where disrupted priors or impaired sensory integration contribute to aberrant self-referential processing in psychosis. Reduced attenuation of self-generated signals, across touch and interoception, may lead to altered salience attribution, a hallmark of psychosis. Future research should explore whether interventions that enhance sensory integration or recalibrate predictive coding can ameliorate symptoms.

## Supplementary information

Supplemental Material

## Data Availability

Analyses code is partly available at osf.io/r3ue7. See supplement for details.
