# Supplementary material for: Altered processing of self-produced sensations in psychosis at cortical and spinal levels
Source: Mol Psychiatry. 2025 Jul 25;30(11):5417–26. doi: 10.1038/s41380-025-03130-w (PMC12532570; doi:10.1038/s41380-025-03130-w)
Supplement: Supplementary file 1 — Supplemental Material [file 41380_2025_3130_MOESM1_ESM.docx]

**Supplementary Material**

**Altered processing of self-produced sensations in psychosis at cortical and spinal levels**

Paula Salamone*^1,2^, Adam Enmalm*^1,2^, Reinoud Kaldewaij^1,2^, Marie Åman^3^, Charlotte Medley^3^, Michal Pietrzak^1,3^, Håkan Olausson^1,4^, Andrea Johansson Capusan^1,3^, Rebecca Boehme^1,2^

*Authors contributed equally

1 Center for Social and Affective Neuroscience, Department of Biomedical and Clinical Sciences, Linköping University, 58185 Linköping, Sweden

2 Center for Medical Imaging and Visualization, Linköping University, 58185 Linköping, Sweden

3 Department for Psychiatry, Linköping University Hospital, 58185 Linköping, Sweden

4 Department of Clinical Neurophysiology, Linköping University Hospital, 58185 Linköping, Sweden

**List of included elements:**

1. Supplementary text: methods, results, discussion
2. Supplementary tables:
   1. S1-4: Demographics of the subsamples per task
   2. S5: Main group effect of fMRI results
   3. S6: Logistic regression analysis
   4. S7: Descriptives for HEP AUC by diagnosis
3. Supplementary figures:
   1. S1: Flow chart of participant attrition
   2. S2: Main effect of group, brain activity difference for patients > controls
4. Supplementary references

**Methods:**

Procedure

Recruitment and inclusion ran from 2021 till 2024. Patients attended three visits, controls two. At visit 1 (only patients), patients were interviewed by a trained psychiatrist using the Brief Psychiatric Rating Scale (BPRS) to assess psychotic symptoms. Controls were interviewed on the phone by a trained staff member using the MINI questionnaire. At Visit 2 (patients and controls), participants underwent neuroimaging at the Center for Medical Imaging and Visualization in Linköping University Hospital, where they performed the self-other touch task. Prior, all participants were instructed to perform the touch inside a dummy scanner. At Visit 3 (patients and controls), participants underwent a neurophysiological evaluation at the Neurophysiology Clinic in Linköping University Hospital. Participants performed the self-other touch task while somatosensory evoked potentials were recorded from the somatosensory cortex and the spinal cord at C6. Additionally, they performed a heartbeat detection task to evaluate interoception at the Center for Social and Affective Neuroscience during EEG recordings. The order of visits 2 and 3 was interchangeable, each lasting 1-2 hours. During one of the visits, touch detection thresholds during the self-other touch task were measured and questionnaires were filled in. Not all participants finished each task (figure S1, tables S1-4). The final sample size was based on previous studies using the same tasks in different populations ^1-4^. During data analyses, outliers >2.5SD from the mean were excluded.

Chlorpromazine equivalents

Current medication was extracted from medical records and chlorpromazine equivalents were calculated in following^5^. In cases where the long-acting injectable (LAI) used by the patient was not included in the conversion tables developed^5^ the LAI was converted to oral form in accordance with tables provided by The American Psychiatric Association^6^ before conversion to chlorpromazine equivalents.

Antidepressant medication

A total of 19 patients were medicated with antidepressant medication. Fluoxetine equivalents were calculated for antidepressant medication^7^. Three patients were medicated with Duloxetine which was not converted into Fluoxetine equivalents. One of these patients was medicated with both Duloxetine and Mirtazapin, and only had the Mirtazapin medication converted and considered for further analysis. Mean Fluoxetine equivalent dose of the 17 considered patients was 31.4 mg (range: 11.1-51.9 mg).

Touch task fMRI

Through a pair of MR-compatible goggles (VisuaStim Digital; Resonance Technologies), participants were prompted on the upcoming condition. The instructions were in Swedish: 1) “Active, please stroke your arm”; 2) “Passive, the experimenter will touch your arm”; and 3) “Active, please stroke the object”. Participants were shown instructions with white text for three seconds (cue phase). After three seconds, the text switched to green, signaling that touch should be initiated (touch-phase). The touching continued for 12 seconds, after which the text was replaced with a plus sign and the touching ended. A 12 second rest period followed. Each condition was repeated 10 times in a random order, for a total task duration of approximately 13.5 minutes.

Data acquisition fMRI

Functional magnetic resonance imaging (fMRI) data was acquired by a 3.0 Tesla scanner (Prisma, Siemens) with a 64-channel head coil. The following settings were used: for T1-weighted anatomical images: repetition time = 2300ms; echo time = 2.36s; flip angle = 8°; Field of view = 288 x 250 mm^2^; Voxel resolution = 0.97 x 0.87 x 0.90 mm^3^, for echo planar images (EPI): repetition time = 1030 ms; echo time = 30 ms; slice thickness = 3 mm; matrix size = 64 x 64; field of view = 192 x 192 mm^2^; in-plane voxel resolution = 3 mm^2^; flip angle = 63°).

MRI data were preprocessed and analyzed in Matlab (MathWorks, MA, USA) using SPM12 (SPM, Wellcome Department of Imaging Neuroscience, UK; http://www.fil.ion.ucl.ac.uk/spm). The preprocessing consisted of motion correction; co-registration of the mean EPI and anatomical (T1) image; segmentation of T1 image and spatial normalization to Montreal Neurological Institute (MNI) template; application of normalization parameters to all EPI volumes; spatial smoothing with an isotropic Gaussian kernel of 6-mm full width at half-maximum.

To control for potential medication effects, covariation with chlorpromazine equivalents were evaluated for each touch condition (self, other, object).

SEP data acquisition

Data were acquired using the NicoletEDX system with an AT2+6 amplifier (Carefusion) and recorded via Synergy 20.0 (Carefusion). Following the standard clinical protocol, the pulses were individually adjusted to induce a small twitch in the thumb yet remain at a tolerable level. The average intensity of stimulation was 11.0 +- 2.6 (Range 5.9-17.3). Recording electrodes were placed at the C6 cervical level, and the C4, CZ, and FZ scalp positions. Skin impedance at the electrode sites was maintained below 8 kΩ. Recordings were referenced online to Fz, bandpass-filtered from 2 Hz to 2 kHz, with an amplifier range of 5 mV and display sensitivity of 20 μV per division.

Baseline-to-peak amplitudes were automatically calculated, with the baseline defined as the point immediately before the average waveform. Peaks were visually inspected and, in case the automatic detection was incorrect, adjusted manually. Data of the three touch conditions were corrected using the baseline condition to control for height and nerve conductance speed.

Heartbeat detection task (HBD) analyses

We deviated from the preregistered analysis plan to calculate a metacognition index based on the difference between objective accuracy and subjective confidence. We changed to the accuracy index because it is based on a newer, less biased method that calculates synchronization accuracy and is uniquely scaled. This index represents accuracy as a mean distance, with zero indicating optimal performance. Increased distance indicates decreased accuracy, complicating a direct comparison between maximum mean distance and minimum confidence scores. This synchronization index reflects the participant's ability to adjust responses to cardiac rhythms without bias from the total number of responses. Therefore, we followed a similar analysis approach, conducting a 2x2 ANOVA and post hoc t-tests with a Tukey correction.

HBD subjective ratings

At the end of each block, participants were asked whether they were sensing their heartbeat and to rate their confidence in their performance across both conditions.

HEP data acquisition

EEG and ECG recordings were acquired using a BIOPAC B-Alert 24-channel system, which comprises 20 active EEG channels, 2 mastoid references, and 2 chest-mounted ECG channels. Data were collected at a sampling rate of 2000 Hz with Acqknowledge software (Biopac) and underwent standard preprocessing as outlined in previous studies^8^.

HEP data processing

The EEG signals were downsampled offline to 250 Hz and bandpass-filtered between 0.5 - 30 μV. The HEP-lab toolbox in Matlab^9^ was used to align ECG events with the continuous EEG signal. To address artifacts related to eye movements, blinks, and cardiac activity, independent component analysis (ICA) and a visual inspection protocol were implemented, based on established methods^1,10^. Any noisy electrodes were excluded from ICA and later interpolated. As early time windows may still carry cardiac field artifacts, only data from 200 to 600 ms post-R-peak from the ECG were analyzed^1,11^. EEG data were divided into epochs spanning -300 to 600 ms and baseline-corrected from -300 to 0 ms relative to the R-peak. Analysis was conducted within a fronto-central region of interest (ROI) including channels Fp1, Fp2, F7, F3, Fz, F4, and F8.

To control for potential confounders, we extracted HEP modulation from the time windows identified as exhibiting significant differences during our initial analysis (main effect of HEP at the group level). This extraction involved calculating the negative area under the curve (AUC) for the mean HEP across the specified time windows (time-window 1: 236-304 ms, time-window 2: 356-500 ms, time-window 3: 520-580 ms) for each participant. We subsequently performed a two-way ANOVA between group and time, revealing a significant effect for group (F(1, 180) = 4.666, p = 0.032) and time (F(2, 180) = 18.574, p < 0.001). Following this, we correlated the average AUC HEP index with potential confounding factors that showed significant differences between groups, specifically medication, body mass index (BMI), and heart rate (HR).

We performed an exploratory analysis to assess HR and heart rate variability (HRV) across groups and conditions with a repeated measures ANOVA. The aim of this analysis was to explore if there was a group by condition interaction that could bias results. ECG data was transformed in Matlab to calculate the R-R interbeat interval that was later analyzed with Kubios software^12^. This is an automated HRV analysis tool for both time and frequency domains, optimized for short intervals. Using an autoregressive algorithm, Kubios computed the power spectrum, distinguishing among high frequency, low frequency , and very low frequency bands. The low/high ratio, a common marker of sympatho/vagal balance, served as an HRV index. For consistency, frequency components were calculated in normalized units (n.u.), representing each component's power relative to total power minus the very low frequency bands.

Combined analyses

To understand whether measures related to symptomatology, we performed three linear regressions on BPRS scores (1) with the experimental measures that differed between groups, (2) with touch-related measures, and (3) with interoception-related measures. The measures included beta-values from fMRI ROIs (STG during self-touch, TPC during other-touch), SEP latency self-other-difference, threshold self-other-difference, HEP AUC during interoception, HBD accuracy difference during interoception. Self-reports were not included. To account for potential outlier influence in the model, we re-ran the regression analysis of touch measures on BPRS total score and negative sub-score with a robust M-estimator with Huber loss in MATLAB. To address potential outliers in our logistic regression predicting group membership (patients vs. controls) from neural predictors, we applied a weighted logistic regression approach in MATLAB. Weights were derived iteratively based on the residuals, approximating robustness by reducing the influence of extreme observations.

We tried to predict group membership from neural measures, using a logistic regression with the regressors: STG ROI during self-touch, TPC ROI during other-touch, SEP latency self-other-difference, HEP AUC during interoception.

To understand the relationship between neural measures of the touch and interoception modalities, we used a linear regression on HEP AUC during interoception with the touch-measures as predictors (beta-values from the STG ROI during self-touch, beta-values from the TPC ROI during other-touch, SEP latency difference between self- and other-condition) and group as a factor.

To control for potential medication effects, chlorpromazine equivalents were correlated with BPRS scores. Furthermore, to control for potential medication and diagnosis effects, we ran five linear regression models on the experimental outcomes that differed between groups (as above) with chlorpromazine equivalents and diagnosis as predictors. To further control for classes of antipsychotic medications, the main analyses were repeated excluding the two patients that received first generation antipsychotics. In addition, the two groups receiving second and third generation antipsychotics were compared on the measures that differed between groups (as above) using two sample t-tests. To control for potential effects of antidepressant medication, the groups that did and did not take antidepressants were compared on the same measures using two sample t-tests. In addition, these measures were correlated with fluoxetine equivalents. To control for illness duration, years since diagnosis were regressed onto the measures that differed between groups.

Code availability

Analyses scripts for fMRI data are available under osf.io/r3ue7. For analysis details on implemented behavioral HBD analysis please see de la Fuente et al. (2019)^25^, Fittipaldi et al. (2020)^26^, for comparison with other behavioral measures of cardiac interoception see Fraile-Vazquez et al. (2025)^27^. For HEP analyses, to support reproducibility, most of the preprocessing pipeline is based on and available through the open-access repository by Fraile et al. (2025)^24^. The pipeline uses modified versions of the EEGLAB and HEPLAB toolboxes, as detailed in the repository. Due to GitHub’s file size limitations (25 MB via the web interface), the full modified EEGLAB+HEPLAB package cannot be uploaded. To access the complete toolboxes, please contact Authors of the repository.

**Results**

fMRI

There were no significant effects for the ROIs other than the ones reported in the main manuscript.

SEPs

*Spinal cord*

There was no significant main effect of condition (𝐹(2,128)= 1.305, 𝑝=0.275, η²=0.006) or group F(1,64)= 0.323, p= 0.572, η²=0.003) on N13 latencies.

Regarding amplitudes in the spinal cord (cervical C6), we found no main effect of condition (𝐹(2,130)= 0.968 𝑝=0.383, η²=0.005) or group F(1,65)= 0.299, p= 0.587, η²=0.003) and the interaction was not significant (F(2,128)= 2.506, p=0.086, η²=0.013).

*Cortex*

At the cortical level, we found no significant differences at Cz or C4 regarding the N20 latencies. The results for Cz were: no main effect of condition (𝐹(2,132)= 0.886, 𝑝= 0.415) , η²=0.004), group F(1,66)= 0.509, p= 0.478, η²=0.005), or interaction (F(2,132)= 1.676, p=0.191, η²=0.008). The results for C4 were: no main effect of condition (𝐹(2,134)= 0.380, 𝑝= 0.685, η²=0.003), group F(1,67)= 0.530, p= 0.469, η²=0.004), or interaction (F(2,134)= 0.634, p=0.532, η²=0.005).

Amplitude of the SEP at the cortical level showed similar effects for both Cz and C4 electrodes. Cz amplitudes showed a main effect of condition (𝐹(2,126)= 13.502, 𝑝< 0.001, η²=0.063), but no effect of group 𝐹(1,63)= 0.142, p= 0.708, η²=0.001) and no interaction (𝐹(2,126)= 0.925, 𝑝= 0.399, η²=0.004). Similarly, C4 amplitudes showed differences for condition (𝐹(2,130)= 11.762, 𝑝< 0.001, η²=0.053), but no effect of group 𝐹(1,65)< 0.001, p= 0.988, η² < 0.001) and no interaction (𝐹(2,130)= 0.230, 𝑝= 0.795, η²=0.001).

The exploratory self-other-difference comparison between groups showed no significant difference for either Cz (t= 0.965, 𝑝= 0.338, η²= -0.055) or C4 (t= 0.012, 𝑝= 0.941, η²=0.003).

Control analysis of SEP stimulation intensity

An independent samples t-test was conducted on stimulation intensity for the SEP. No significant difference between groups were found (t(68)=0.938, p=0.351, d = 0.224).

Touch detection thresholds

There were no significant results for condition (𝐹(2,128)= 0.218, 𝑝= 0.642, η²=0.002), group 𝐹(1, 128)= 0.033, 𝑝= 0.856, η²<0.001), or interaction 𝐹(1, 128)= 0.584, 𝑝= 0.446, η²=0.005).

HBD – exteroception accuracy and subjective ratings

There were no significant differences between groups for the exteroceptive condition (t=-0.374, p= 0.982). Both groups had higher accuracy during exteroception compared to interoception (controls: t= -7.462, p< 0.001; patients: t= -10.244, p< 0.001).

Confidence score analysis revealed a main effect of condition (𝐹(1,132)= 87.039, 𝑝< 0.001), but there was no effect of group (𝐹(1,132)= 1.776, 𝑝< 0.185), or interaction (𝐹(1,132)= 0.197, 𝑝= 0.658). Posthoc assessment indicates that both controls (t=6.911, p< 0.001) and patients (t= 6.283, p< 0.001) rated confidence higher for exteroception, as expected for a clearly perceivable stimulus.

Similar results were found for the detection of the signals: there was a main effect of condition (𝐹(1,132)= 99.029, 𝑝< 0.001), but there was no effect of group (𝐹(1,132)= 1.283, 𝑝< 0.259), or interaction (𝐹(1,132)= 0.519, 𝑝= 0.519). Posthoc assessment indicates that both controls (t=7.494, p< 0.001) and patients (t= 6.579, p< 0.001) detected the exteroceptive signals more clearly.

HEP

Within groups, we found that the controls displayed the expected HEP modulation, with higher amplitudes during the interoceptive than during the exteroceptive condition in three time-windows (time-window 1: 424-452 ms, time-window 2: 508-540 ms, time-window 3: 580-600 ms, Figure 4B).

*Heartrate*

We found that there was a main effect of condition (F (1, 63)= 8.610, p= 0.005) and group (F (1, 63)= 5.586, p= 0.021). Both groups displayed a lower HR during interoception (patients mean= 81.120, SD= 13.672; controls mean= 73.182, SD= 12.670) compared to the exteroceptive condition (patients mean= 81.862, SD= 13.701; controls mean= 74.482, SD= 12.291). Controls had lower HR regardless of condition, but there were no interaction effects (F (1, 63)= 0.642, p= 0.426) that could be potentially confounding the significant differences found in each condition.

Regarding HRV, we found a main effect of condition: HRV was lower during interoception (F (1, 63)= 7.904, p= 0.007) in both groups. However, we did not find any group (F (1, 63)= 1.056, p= 0.308) or interaction effects (F (1, 63)= 0.619, p= 0.434) that could potentially confound our results.

Relation to symptoms

The models including all measures (F(6,19)=1.93, p=0.146) and the interoception measures only were not significant (F(2,28)=2.48, p=0.103).

We further explored the three main subscales of the BPRS. Touch-related measures significantly predicted negative symptoms (F(4, 24) = 3.44, p = 0.026, adjusted R² = 0.396), but not affective symptoms (F(4,24) = 2.56, p = 0.068) or positive symptoms (F(4,24) = 0.59, p = 0.68).

An additional exploratory analysis using the interoception-measures found no relation to BPRS subscales (positive: F(2,26) = 1.42, p = 0.26; negative: F(2,26) = 1.64, p = 0.21, affective: F(2,26) = 1.8, p = 0.19).

To account for the potential influence of outliers in the data, we conducted a robust linear regression control analyses for the touch-measures regression on BPRS total using an M-estimator with Huber loss. The model yielded the following coefficients (with intercept): Intercept = 33.83, STG activation (self-touch) = 12.32 (p = 0.1016), TPC activation (other-touch) = -4.60 (p = 0.1869), SEP latency difference = 3.79 (p = 0.0929), Touch threshold difference = -0.98 (p = 0.0837). These results indicate a similar pattern to the standard regression, with none of the predictors reaching conventional statistical significance under robust estimation. However, the pattern remained comparable, and several predictors showed trends toward significance. Observation weights (ranging from 0.80 to 1.00) suggest that no single data point overly influenced the model. This supports the robustness of the associations, while also reflecting the expected interindividual variability in our clinically heterogeneous and relatively small patient sample.

For the BPRS negative symptom scale, the model yielded the following coefficients (with intercept): Intercept = 6.50, STG activation (self-touch) = 3.63 (p = 0.1392), TPC activation (other-touch) = -2.01 (p = 0.0839), SEP latency difference = 0.85 (p = 0.2445), Touch threshold difference = -0.41 (p = 0.0314). Touch threshold difference significantly predicted negative symptom severity (p = 0.0314), suggesting a robust link between somatosensory alterations and clinical presentation. While other predictors did not reach significance, TPC activation showed a trend. The range of observation weights (0.19–1.00) indicates moderate interindividual variability, with a few down-weighted observations.A logistic regression with Huber-loss like weighing of outlier supported our findings: The model yielded significant predictive value for the STG activation (self-touch) (coefficient = 5.72, p = 0.004), indicating higher activation in this region increased the odds of being classified as a patient.

Control analyses for medication and diagnosis

*Chlorpromazine equivalents*

There was no correlation with chlorpromazine equivalents during any of the touch conditions at the whole brain level (p (FWE-corrected) > 0.05).The HEP results in patients were not associated with their medication intake (rho= -0.033, p= 0.864). Additionally, HEP modulation in the total sample was not associated with BMI (rho= 0.082, p= 0.531). HEP was associated with HR (rho= 0.459, p< 0.001), but not HRV (rho= 0.003, p= 0.982). Although HR can influence overall HEP, we found similar correlation for both groups (C: rho= 0.407, p= 0.023, P: rho= 0.463, p= 0.012). Therefore, HR might be related to HEP, but it does not explain the group differences.

There was no correlation between chlorpromazine equivalents and BPRS total score (r = -0.163, p = 0.349) or the subscores (positive: r = 0.028, p = 0.349, negative: r = -0.251, p = 0.146, affective: r = -0.188, p = 0.31).

Chlorpromazine equivalents and diagnosis did not predict most of the measures that differed between groups (self-touch in STG: F(6,28)=0.159, p=0.975, other-touch in TPC: F(6,28)=0.056, p=0.998, SEP: F(6,28)=0.574, p=0.748, thresholds: F(6,28)=1.073, p=0.399, HBD accuracy: F(6,25)=0.913, p=0.502). The model predicting HEP AUC during interoception was significant (F6,23)=2.76, p=0.036, adjusted R² = 0.267). This was not driven by medication (t=0.782, p=0.442), but by an effect from diagnosis, where non-specific non-organic psychosis differed significantly (t=-2.784, p=0.011). HEP AUC was larger (more negative) in this group compared to the schizophrenia group (Table S7). This diagnosis is typically given while the patient is still under investigation and will often be updated to schizophrenia or schizoaffective disorder later. Descriptively, this group had the shortest years since diagnosis (2.43±2.573; schizophrenia: 6.5±6.13; schizoaffective: 3.9±3.1; delusional disorder: 4.67±3.22, paranoid schizophrenia: 6, acute schizophrenia-like psychosis: 8). However, group sizes per diagnosis were very small and do not allow for a conclusive interpretation. An exploratory model regressing years since diagnosis onto HEP AUC was not significant (F(1,28)=0.094, p=0.761).

*First generation antipsychotic medication control*

To assess the robustness of the main findings, we repeated the main analysis excluding the two patients treated with first-generation antipsychotics.

fMRI: One of the patients with first generation antipsychotics had not participated in fMRI. When excluding the other one patient, results in the main effect remained largely the same. When excluding the one participant from the two sample t-tests per condition, the analyses showed the same peak, but were slightly above significance threshold [for the STG small volume: *p*_FWE(SVC)_=0.052, MNI_xyz_=48,-26,14; *p*_FWE(SVC)_=0.070, MNI_xyz_=42,-30,16; for the TPC small volume: (p_FWE(SVC)_=0.080, MNI_xyz_=58,-26,22). For the extracted beta-values from STG and TPC, the first generation patient was not an outlier.

Spinal cord latency (N13): After removing two participants with first generation antipsychotics, the condition x group interaction, although slightly stronger, showed same pattern of results (F(2,124) = 3.412, p = 0.036, η² = 0.016). Similarly, follow-up comparisons confirmed that patients continued to show significantly smaller latency differences between self-other-touch difference (U = 757, p = 0.014, rank biserial r = 0.349), consistent with the main findings.

Touch thresholds: After removing two participants with first generation antipsychotics, the two-way ANOVA revealed no significant main effects of condition (F(1,124) = 0.391, p = 0.533, η² = 0.003), group (F(1,124) = 0.099, p = 0.899, η²< 0.001), or interaction (F(1,124) = 0.323, p = 0.571, η² = 0.003). Exploratory analysis indicated a trend toward a group difference in threshold modulation between self- and other-touch (U = 663.0, p = 0.068, rank biserial r = 0.263).

HBD: One of the patients with first generation antipsychotics had not been included in the behavioral HBD analysis, none were included in the HEP analysis. After controlling by excluding one participant from the behavioral analysis, the main effect of group remained similar: 𝐹(1,125)=6.462, p=0.012. η² = 0.022, group x condition: 𝐹(1,127)=4.037, p=0.047, η² = 0.014). Post hoc test confirmed a significant group difference in the interoceptive condition (t=-2,381, p=0.02, Cohen’s d = –0.595), where patients displayed lower accuracy.

When excluding the two patients on first generation antipsychotics, the results on the relationship between our measures and BPRS and on predicting group did not change. The statistics without these two patients were as follows: Touch-related measures predicting BPRS total scores (F(4,24)=2.962, p=0.045, adjusted R²=0.246), with predictors touch threshold difference between self- and other-touch (t=-2.112, p=0.047), STG activity during self-touch (t=2.11, p=0.048 [this is just above significance threshold in main analysis]), SEP spinal latency difference (t=1.771, p=0.092). Predicting group membership: (X^2^=19.37, p<0.001), predictor STG activation during self-touch (Wald statistic=7.6, p=0.006, Odds Ratio 57.96). The model explained 42.1% of the variance (Nagelkerke R^2^) and predicted 70.6% of the cases correctly.

*Second and third generation antipsychotic medication control*

We further compared the measures between patients receiving second and third generation antipsychotics. There was no significant difference between groups (self-touch in STG: t=-0.07, p=0.945, SEP: t=-0.342, p=0.735, thresholds: t=0.184, p=0.856, HBD accuracy: t=-0.2, p=0.843, HEP AUC: t=-0.23, p=0.82), except for other-touch in TPC, t=2.6, p=0.015. The latter was driven by higher beta-parameter estimates in the temporoparietal cluster during other-touch in the group receiving second generation antipsychotics (second: 0.786±0.36, third: 0.435±0.326), but does not survive correction for multiple comparison and the subgroups are small (second n=17, third n=11). We also compared BPRS subscores between the two groups and found a difference for the negative symptoms (t=-2.82, p=0.008, positive: t=-1.29, p=0.208, affective: t=-0.32, p=0.75), which were higher in the group receiving third generation antipsychotics (second: 5.1±2.1, third: 7.5±2.6). Beta-parameter estimates in the TPC in turn correlated with the negative symptom subscale (r=-0.406, p=0.032).

*Antidepressants control analyses*

There was no difference for any measure between the groups receiving antidepressants and not receiving antidepressants (self-touch in STG: t=-0.89, p=0.28, other-touch in TPC SEP: t=-0.2, p=0.84, SEP: t=0.89, p=0.4, thresholds: t=-1.19, p=0.25, HBD accuracy: t=0.96, p=0.34, HEP AUC: t=0.01, p=0.99). Fluoxetine equivalents did not correlate with any of the measures (r’s<0.36, p’s>0.14).

*Control analyses for illness duration*

There was no effect of years since diagnosis on any of the measures that differed between groups (antidepressants (self-touch in STG: F(1,25)=2.35, p=0.13, other-touch in TPC SEP: f(1,25)=0.15, p=0.7, SEP: F(1,31)=1.6, p=0.22, thresholds: F(1,27)=0.33, p=0.57, HBD accuracy: F(1,29)=0.72, p=0.4, HEP AUC: F(1,27)=0.09, p=0.76).

Touch-interoception relationship

Neural measures of touch did not predict HEP during interoception (F(5,48) = 1.1, p = 0.37).

**Discussion**

Control analyses for regressions

Robust regression analyses using an M-estimator with Huber loss largely confirmed our main findings, showing comparable effect sizes despite none reaching conventional significance for total BPRS scores. Importantly, observation weights indicated no single data point unduly influenced results, highlighting that individual “outliers” in this clinically heterogeneous and small sample likely represent meaningful variance rather than noise.

For negative symptoms, touch threshold difference significantly predicted severity, reinforcing the link between somatosensory alterations and clinical presentation. Robust logistic regression similarly confirmed superior temporal gyrus activation during self-touch as a significant predictor of group status.

These results underscore the stability of our findings while acknowledging that variability in this patient group reflects relevant individual differences rather than spurious outliers.

Forward-model and social touch

Regarding the attenuation of self-produced touch sensations, it is important to mention that the forward model, that suggests a simple efference copy of the motor command to be responsible for the attenuation, appears too simple in the light of recent findings. Studies have elaborated this model and found that movement alone does not suffice attenuation^13^, indicating the need for intent for self-touch to be attenuated. Inversely, the incorrect self-attribution of touch induces attenuation even in the absence of movement^14^. The intent can be considered the sense of agency, i.e. being the cause of an action^15^. People with schizophrenia reportedly experience a decreased sense of agency^16^, particularly those with primarily negative symptoms^17^. This fits with our finding that neural measures of touch were related to negative and affective symptoms, but it contrasts with the earlier results of lowered sensory attenuation to be present mainly in those with positive symptomatology^18^.

For other-touch, patients showed increased activity in a temporoparietal cluster that overlaps with the secondary somatosensory cortex, posterior insula, and temporo-parietal junction. This region of interest was functionally defined on our previous findings on self-other-touch processing changes during a pharmacological manipulation of the sense of self using ketamine^19^. We found a reduction of other-related activity in this area during the ketamine session – the opposite from our here reported findings in schizophrenia. This is especially of interest because ketamine has been suggested as a pharmacological model for schizophrenia before^20,21^. A potential explanation for this disparity could be that ketamine can be understood as a model for acute or early stage psychosis, while our patients had been diagnosed for several years and were medicated. However, it is also possible that our findings indicate a different underlying mechanism in schizophrenia than during ketamine administration. Finally, a more basic explanation could be that schizophrenia patients experience less social affective touch in their daily life than controls^22^ and it has been shown that touch deprivation relates to social touch perception^23^. Therefore, an increased neural response to social touch in schizophrenia might simply indicate that this stimulus is more salient, relevant, or surprising. We did however not find increased responses to social touch in its primary processing areas, the primary somatosensory cortex and the insula.

**Tables**:

**Table S1. Sample who completed the heartbeat tapping task**

| **Basic Demographics** | Control | Patient | Test variable | | Significance | Effect size |  |
| --- | --- | --- | --- | --- | --- | --- | --- |
| Gender | 15F/19M | 14F/20M | χ^2^ (1) = 0.060 | p = 0.806 | | V = 0.030 |  |
| Age | 35.5 (9.4) | 35.6 (7.5) | t (66) = -0.043 | p = 0.966 | | d = 0.020 |  |
| BMI | 24.3 (4.3) | 29.4 (6.0) | t (66) = -4.039 | p < 0.001** | | d = -0.928 |  |
|  |  |  |  |  | |  |  |
| **Handedness** |  |  | χ^2^ (2) = 4.410 | p = 0.110 | | V = 0.255 |  |
| Right | 33 (97.1%) | 28 (82.4%) |  |  | |  |  |
| Left | 0 (0%) | 3 (8.8%) |  |  | |  |  |
| Ambidextrous | 1 (2.9%) | 3 (8.8%) |  |  | |  |  |
|  |  |  |  |  | |  |  |
| **Education** |  |  | χ^2^ (2) = 7.111 | p = 0.029* | | V = 0.323 |  |
| Primary | 0 (0%) | 5 (14.7%) |  |  | |  |  |
| Secondary | 12 (35.3%) | 15 (44.1%) |  |  | |  |  |
| Tertiary | 22 (64.7%) | 14 (41.2%) |  |  | |  |  |
|  |  |  |  |  | |  |  |
| **Occupation** |  |  | χ^2^ (2) = 24.571 | p < 0.001** | | V = 0.601 |  |
| Unemployed / sick | 0 (0%) | 4 (11.8%) |  |  | |  |  |
| Student | 10 (29.4%) | 18 (52.9%) |  |  | |  |  |
| Working | 24 (70.6%) | 12 (35.3%) |  |  | |  |  |
|  |  |  |  |  | |  |  |
| **Questionnaires** |  |  |  |  | |  |  |
| MAIA | 101.7 (21.8) | 87.6 (18.6) | t (66) = 2.872 | p = 0.005* | | d = 0.696 |  |
| MAIA: Noticing | 3.6 (0.9) | 3.2 (0.9) | t (66) = 2.068 | p = 0.043 | | d = 0.502 |  |
| MAIA: Non-distracting | 2.5 (0.8) | 2.1 (0.8) | t (66) = 1.823 | p = 0.073 | | d = 0.442 |  |
| MAIA: Non-worrying | 3.1 (0.9) | 2.3 (1.2) | t (66) = 3.098 | p = 0.003* | | d = 0.751 |  |
| MAIA: Attention regulation | 3.2 (0.9) | 2.9 (0.9) | t (66) = 1.449 | p = 0.152 | | d = 0.351 |  |
| MAIA: Emotional awareness | 3.4 (0.9) | 3.0 (1.0) | t (66) = 1.721 | p = 0.090 | | d = 0.417 |  |
| MAIA: Self-regulation | 3.0 (1.1) | 2.5 (1.1) | t (66) = 1.851 | p = 0.069 | | d = 0.449 |  |
| MAIA: Bodily listening | 2.5 (1.1) | 2.5 (1.1) | t (66) > 0.001 | p = 1.000 | | d < 0.001 |  |
| MAIA: Trusting | 3.8 (1.0) | 3.0 (1.4) | U = 783.5 | p = 0.011 | | r_rb_ = 0.356 |  |
| STQ | 27.3 (9.2) | 34.0 (14.2) | U = 429.5 | p = 0.069 | | r_rb_ = -0.257 |  |
| EQ | 46.3 (13.1) | 41.3 (13.0) | t (66) = 1.590 | p = 0.117 | | d = 0.386 |  |
| SP: Low Registration | 28.6 (5.6) | 34.0 (7.6) | U = 322.0 | p = 0.002* | | r_rb_ = -0.443 |  |
| SP: Sensation Seeking | 42.7 (5.9) | 41.2 (7.6) | t (66) = 0.928 | p = 0.357 | | d = 0.225 |  |
| SP: Sensation Sensitivity | 33.4 (5.4) | 37.7 (9.5) | U = 402.5 | p = 0.032 | | r_rb_ = -0.304 |  |
| SP: Sensation Avoiding | 36.3 (7.0) | 40.9 (9.2) | t (66) = -2.296 | p = 0.025 | | d = -0.557 |  |
| SP: Touch processing | 29.1 (3.8) | 30.5 (6.7) | U = 495.0 | p = 0.310 | | r_rb_ = -0.144 |  |
| *Note. * Significant after correction for multiple comparisons using Bonferroni-Holms* | | | | | | | |

**Table S2. Sample who completed the somatosensory evoked potentials**

| **Basic Demographics** | Control | Patient | Test variable | Significance | Effect size |  |
| --- | --- | --- | --- | --- | --- | --- |
| Gender | 15F/20M | 14F/20M | x^2^ (1) = 0.060, | p = 0.806 | V = 0.017 |  |
| Age | 35.7 (9.3) | 35.5 (7.5) | t (66) = -0.043, | p = 0.966 | d = 0.019 |  |
| BMI | 24.3 (4.3) | 29 (6.2) | t (66) = -4.039, | p < 0.001* | d = -0.947 |  |
|  |  |  |  |  |  |  |
| **Handedness** |  |  | x^2^ (2) = 4.410 | p = 0.110 | V = 0.257 |  |
| Right | 34 (97.1%) | 28 (82.4%) |  |  |  |  |
| Left | 0 (0%) | 3 (8.8%) |  |  |  |  |
| Ambidextrous | 1 (2.9%) | 3 (8.8%) |  |  |  |  |
|  |  |  |  |  |  |  |
| **Education** |  |  | x^2^ (2) = 7.802, | p = 0.020* | V = 0.336 |  |
| Primary | 0 (0%) | 6 (17.6%) |  |  |  |  |
| Secondary | 13 (37.1%) | 14 (41.2%) |  |  |  |  |
| Tertiary | 22 (65.9%) | 14 (41.2%) |  |  |  |  |
|  |  |  |  |  |  |  |
| **Occupation** |  |  | x^2^ (2) = 23.225 | p < 0.001* | V = 0.580 |  |
| Unemployed / sick | 0 (0%) | 17 (50.0%) |  |  |  |  |
| Student | 10 (28.6%) | 5 (14.7%) |  |  |  |  |
| Working | 25 (71.4%) | 12 (35.3%) |  |  |  |  |
|  |  |  |  |  |  |  |
| **Questionnaires** |  |  |  |  |  |  |
| MAIA | 101.5 (21.5) | 87.2 (18.6) | t (67) = 2.958 | p = 0.004* | d = 0.712 |  |
| MAIA: Noticing | 3.6 (0.8) | 3.2 (0.9) | t (67) = 2.012 | p = 0.048 | d = 0.485 |  |
| MAIA: Non-distracting | 2.4 (0.8) | 2.1 (0.8) | t (67) = 1.854 | p = 0.068 | d = 0.446 |  |
| MAIA: Non-worrying | 3.1 (0.9) | 2.3 (1.2) | t (67) = 3.136 | p = 0.003* | d = 0.755 |  |
| MAIA: Attention regulation | 3.2 (0.9) | 2.9 (1.0) | t (67) = 1.199 | p = 0.235 | d = 0.289 |  |
| MAIA: Emotional awareness | 3.4 (0.9) | 2.9 (1.1) | t (67) = 2.057 | p = 0.044 | d = 0.495 |  |
| MAIA: Self-regulation | 3.0 (1.0) | 2.4 (1.1) | t (67) = 2.029 | p = 0.046 | d = 0.489 |  |
| MAIA: Bodily listening | 2.5 (1.1) | 2.5 (1.2) | t (67) = 0.157 | p = 0.876 | d = 0.038 |  |
| MAIA: Trusting | 3.8 (1.0) | 3.0 (1.4) | U = 790.0 | p = 0.018 | r_rb_ = 0.328 |  |
| STQ | 37.2 (9.1) | 44.4 (14.1) | U = 419.0 | p = 0.035 | r_rb_ = -0.296 |  |
| EQ | 46.1 (13.0) | 41.7 (12.9) | t (67) = 1.413 | p = 0.162 | d = 0.340 |  |
| SP: Low Registration | 28.5 (5.6) | 33.9 (7.6) | U = 330.5 | p = 0.001* | r_rb_ = -0.445 |  |
| SP: Sensation Seeking | 42.6 ( 5.9) | 40.9 (7.5) | t (67) = 1.044 | p = 0.300 | d = 0.251 |  |
| SP: Sensation Sensitivity | 33.1 (5.6) | 37.9 (9.7) | U = 399.0 | p = 0.019 | r_rb_ = -0.511 |  |
| SP: Sensation Avoiding | 36.1 (7.1) | 41.1 (9.0) | t (67) = -2.581 | p = 0.012 | d = -0.621 |  |
| SP: Touch processing | 28.9 (4.1) | 30.4 (6.7) | U = 505.0 | p = 0.282 | r_rb_ = -0.151 |  |
| *Note. * Significant after correction for multiple comparisons using Bonferroni-Holms* | | | | | | |

**Table S3. Sample who completed the touch threshold task.**

| **Basic Demographics** | Control | Patient | Test variable | Significance | Effect size |  |
| --- | --- | --- | --- | --- | --- | --- |
| Gender | 15F/20M | 14F/20M | x^2^ (1) = 0.035 | p = 0.851 | V = 0.023 |  |
| Age | 35.7 (9.3) | 35.7 (7.3) | U = 550.5 | p = 0.923 | d = -0.002 |  |
| BMI | 24.3 (4.3) | 29 (6.2) | t (64) = -3.846 | p < 0.001* | d = -0.949 |  |
|  |  |  |  |  |  |  |
| **Handedness** |  |  | x^2^ (2) = 3.838 | p = 0.147 | V = 0.241 |  |
| Right | 34 (97.1%) | 26 (83.9%) |  |  |  |  |
| Left | 0 (0%) | 2 (6.4%) |  |  |  |  |
| Ambidextrous | 1 (2.9%) | 3 (9.7%) |  |  |  |  |
|  |  |  |  |  |  |  |
| **Education** |  |  | x^2^ (2) = 8.731 | p = 0.013* | V = 0.364 |  |
| Primary | 0 (0%) | 6 (19.4%) |  |  |  |  |
| Secondary | 13 (37.1%) | 13 (41.9%) |  |  |  |  |
| Tertiary | 22 (62.9%) | 12 (38.7%) |  |  |  |  |
|  |  |  |  |  |  |  |
| **Occupation** |  |  | x^2^ (2) = 21.949 | p < 0.001* | V = 0.577 |  |
| Unemployed / sick | 0 (0%) | 15 (48.4%) |  |  |  |  |
| Student | 10 (28.6%) | 5 (16.1%) |  |  |  |  |
| Working | 25 (71.4%) | 11 (35.5%) |  |  |  |  |
|  |  |  |  |  |  |  |
| **Questionnaires** |  |  |  |  |  |  |
| MAIA | 101.5 (21.5) | 85.8 (18.6) | t (64) = 3.167 | p = 0.002* | d = 0.781 |  |
| MAIA: Noticing | 3.6 (0.8) | 3.2 (0.9) | t (64) = 1.926 | p = 0.059 | d = 0.475 |  |
| MAIA: Non-distracting | 2.4 (0.8) | 2.1 (0.8) | t (64) = 1.768 | p = 0.082 | d = 0.436 |  |
| MAIA: Non-worrying | 3.1 (0.9) | 2.4 (1.2) | t (64) = 2.735 | p = 0.008 | d = 0.675 |  |
| MAIA: Attention regulation | 3.2 (0.9) | 2.9 (1.0) | t (64) = 1.250 | p = 0.216 | d = 0.308 |  |
| MAIA: Emotional awareness | 3.4 (0.9) | 2.9 (1.2) | t (64) = 2.108 | p = 0.039 | d = 0.520 |  |
| MAIA: Self-regulation | 3.0 (1.0) | 2.3 (1.0) | t (64) = 2.594 | p = 0.012 | d = 0.640 |  |
| MAIA: Bodily listening | 2.5 (1.1) | 2.3 (1.1) | t (64) = 0.737 | p = 0.464 | d = 0.182 |  |
| MAIA: Trusting | 3.8 (1.0) | 2.9 (1.3) | U = 753.5 | p = 0.006* | r_rb_ = 0.389 |  |
| STQ | 37.2 (9.1) | 45.0 (14.4) | U = 371.0 | p = 0.028 | r_rb_ = -0.224 |  |
| EQ | 46.1 (13.0) | 41.4 (12.8) | t (64) = 1.467 | p = 0.147 | d = 0.362 |  |
| SP: Low Registration | 28.5 (5.6) | 34.6 (7.3) | U = 276.0 | p < 0.001* | r_rb_ = -0.491 |  |
| SP: Sensation Seeking | 42.6 (5.9) | 41.1 (7.7) | t (64) = 0.864 | p = 0.391 | d = 0.213 |  |
| SP: Sensation Sensitivity | 33.1 (5.6) | 38.9 (9.3) | U = 322.5 | p = 0.005* | r_rb_ = -0.406 |  |
| SP: Sensation Avoiding | 36.1 (7.1) | 41.3 (9.4) | t (64) = -2.589 | p = 0.012 | d = -0.638 |  |
| SP: Touch processing | 28.6 (4.1) | 31.2 (6.1) | U = 422.5 | p = 0.124 | r_rb_ = -0.221 |  |
| *Note. * Significant after correction for multiple comparisons using Bonferroni-Holms* | | | | | |  |

**Table S4. Sample who completed the functional magnetic resonance imaging.**

| **Basic Demographics** | Control | Patient | Test variable | Significance | Effect size |
| --- | --- | --- | --- | --- | --- |
| Gender | 11F/20M | 11F/17M | x^2^ (1) = 0.091 | p = 0.763 | V = 0.039 |
| Age | 34.8 (9.1) | 35.5 (7.7) | t (57) = -0.264 | p = 0.793 | d = -0.069 |
| BMI | 24.0 (4.1) | 28.9 (6.0) | t (57) = -3.680 | p < 0.001* | d = -0.959 |
|  |  |  |  |  |  |
| **Handedness** |  |  | x^2^ (2) = 5.091 | p = 0.078 | V = 0.294 |
| Right | 30 (96.7%) | 22 (78.6%) |  |  |  |
| Left | 0 (0%) | 3 (10.7%) |  |  |  |
| Ambidextrous | 1 (3.3%) | 3 (10.7%) |  |  |  |
|  |  |  |  |  |  |
| **Education** |  |  | x^2^ (2) = 8.776 | p = 0.012* | V = 0.401 |
| Primary | 0 (0%) | 6 (20.7%) |  |  |  |
| Secondary | 12 (38.7%) | 13 (44.8%) |  |  |  |
| Tertiary | 19 (61.3%) | 10 (34.5%) |  |  |  |
|  |  |  |  |  |  |
| **Occupation** |  |  | x^2^ (2) = 19.652 | p < 0.001* | V = 0.561 |
| Unemployed / sick | 0 (0%) | 14 (48.3%) |  |  |  |
| Student | 10 (32.3%) | 4 (13.8%) |  |  |  |
| Working | 21 (67.7%) | 11 (37.9%) |  |  |  |
|  |  |  |  |  |  |
| **Questionnaires** |  |  |  |  |  |
| MAIA | 99.3 (21.4) | 88.3 (19.1) | t (57) = 2.088 | p = 0.041 | d = 0.544 |
| MAIA: Noticing | 3.6 (0.8) | 3.3 (0.8) | t (57) = 1.458 | p = 0.150 | d = 0.380 |
| MAIA: Non-distracting | 2.4 (0.8) | 1.9 (0.8) | t (57) = 2.079 | p = 0.042 | d = 0.542 |
| MAIA: Non-worrying | 3.1 (0.9) | 2.4 (1.3) | t (57) =2.322 | p = 0.024 | d = 0.605 |
| MAIA: Attention regulation | 3.1 (0.9) | 3.0 (0.9) | t (57) = 0.667 | p = 0.508 | d = 0.174 |
| MAIA: Emotional awareness | 3.4 (0.9) | 3.0 (1.1) | t (57) = 1.459 | p = 0.150 | d = 0.380 |
| MAIA: Self-regulation | 2.9 (1.1) | 2.4 (1.2) | t (57) = 1.672 | p = 0.100 | d = 0.436 |
| MAIA: Bodily listening | 2.4 (1.0) | 2.5 (1.3) | t (57) = -0.529 | p = 0.599 | d = -0.138 |
| MAIA: Trusting | 3.8 (1.0) | 3.1 (1.3) | U = 546.0 | p = 0.087 | r_rb_ = 0.258 |
| STQ | 37.6 (9.3) | 44.6 (14.0) | U = 314.0 | p = 0.069 | r_rb_ = -0.276 |
| EQ | 45.5 (13.5) | 40.7 (12.7) | t (57) = 1.397 | p = 0.168 | d = 0.364 |
| SP: Low Registration | 28.3 (5.6) | 34.6 (7.2) | t (57) = -3.766 | p < 0.001* | d = -0.982 |
| SP: Sensation Seeking | 42.3 (5.3) | 40.7 (7.8) | t (57) = 0.923 | p = 0.360 | d = 0.241 |
| SP: Sensation Sensitivity | 33.0 (5.7) | 38.2 (9.5) | U = 269.5 | p = 0.013 | r_rb_ = 0.212 |
| SP: Sensation Avoiding | 36.0 (7.2) | 41.6 (9.5) | t (57) = -2.560 | p = 0.013 | d = -0.667 |
| SP: Touch processing | 29.2 (4.1) | 30.1 (6.8) | U = 401.0 | p = 0.069 | r_rb_ = -0.076 |
| *Note. * Significant after correction for multiple comparisons using Bonferroni-Holms* | | | | | |

**Table S5: Main effect of group.**

| **Patients > Controls** |  |  |  |  |  |  |  |
| --- | --- | --- | --- | --- | --- | --- | --- |
| **Region** | ***k*** | **L/R** | **x** | **y** | **z** | ***t*** | ***p(FWE)*** |
| Secondary Visual cortex | 564 | R | 16 | -92 | 12 | 14.87 | <0.001 |
|  |  |  | 26 | -86 | 14 | 11.61 | <0.001 |
|  |  |  | 38 | -84 | 4 | 9.22 | <0.001 |
|  | 23 | L | -24 | -88 | 2 | 6.78 | <0.001 |
| Medial temporal gyrus | 507 | R | 54 | -50 | 6 | 8.98 | <0.001 |
|  |  |  | 42 | -66 | 8 | 7.90 | <0.001 |
|  |  |  | 52 | -64 | 2 | 5.70 | <0.001 |
| Fusiform gyri | 345 | R | 28 | -66 | -12 | 8.73 | <0.001 |
|  |  |  | 30 | -78 | -16 | 7.95 | <0.001 |
|  |  |  | 26 | -82 | -10 | 7.52 | <0.001 |
| Primary Somatosensory cortex | 1058 | R | 50 | -20 | 40 | 8.33 | <0.001 |
|  |  |  | 48 | -28 | 12 | 8.25 | <0.001 |
|  |  |  | 60 | -44 | 26 | 8.07 | <0.001 |
|  | 31 | R | 32 | -46 | 66 | 7.15 | <0.001 |
|  | 86 | L | -10 | -34 | 70 | 6.95 | <0.001 |
|  |  |  | 0 | -36 | 64 | 5.94 | 0.001 |
|  |  |  | -10 | -44 | 72 | 5.39 | 0.010 |
| Premotor cortex | 162 | R | 44 | -8 | 56 | 7.90 | <0.001 |
|  |  |  | 38 | -10 | 64 | 7.35 | <0.001 |
|  | 30 | R | 52 | 2 | 32 | 6.41 | <0.001 |
|  |  |  | 36 | -18 | 66 | 7.33 | <0.001 |
| Angular gyrus | 441 | R | -54 | -44 | 26 | 7.77 | <0.001 |
|  |  |  | -46 | -30 | 10 | 7.09 | <0.001 |
|  |  |  | -54 | -32 | 14 | 7.02 | <0.001 |
| Supramarginal gyrus | 114 | R | 36 | -40 | 52 | 7.65 | <0.001 |
|  |  |  | 42 | -36 | 62 | 6.01 | 0.001 |
|  |  |  | 44 | -32 | 54 | 5.29 | 0.014 |
|  | 69 | L | -52 | -30 | 44 | 7.17 | <0.001 |
|  |  |  | -56 | -36 | 48 | 5.77 | 0.002 |
| Secondary Somatosensory cortex | 49 | R | 10 | -38 | 58 | 7.44 | <0.001 |
| Dorsal Posterior cingulate cortex | 454 | R | 4 | -26 | 46 | 7.21 | <0.001 |
|  |  |  | 2 | -12 | 42 | 6.57 | <0.001 |
|  |  |  | 2 | -8 | 34 | 6.54 | <0.001 |
| Visual cortex | 119 | L | -46 | -66 | 10 | 7.13 | <0.001 |
| Operculum | 77 | R | 54 | 4 | 0 | 6.50 | <0.001 |
| Primary Motor cortex | 40 | R | 56 | -2 | 16 | 6.37 | <0.001 |
| Insula | 35 | R | 40 | -10 | -14 | 6.17 | <0.001 |

| **Controls > Patients** |  |  |  |  |  |  |  |
| --- | --- | --- | --- | --- | --- | --- | --- |
| **Region** | **k** | **L/R** | **x** | **y** | **z** | **t** | **p(FWE)** |
| Secondary visual cortex | 99 | R | 12 | -80 | -2 | 12.72 | <0.001 |
|  | 517 | L | -12 | -82 | -12 | 10.55 | <0.001 |
|  |  |  | -10 | -78 | 4 | 8.39 | <0.001 |
|  |  |  | -10 | -94 | 2 | 8.36 | <0.001 |
| Calcarine cortex | 148 | R | 6 | -86 | 8 | 9.13 | <0.001 |
|  |  |  | 16 | -80 | 10 | 7.39 | <0.001 |
| Cuneus | 26 | R | 10 | -90 | 22 | 7.16 | <0.001 |

**Table S6.: Logistic regression analysis of neural measures on group membership**

| Independent variable | Coefficient estimate | Standard error | Z | P | Odds Ratio |
| --- | --- | --- | --- | --- | --- |
| Intercept | -0.47 | 0.788 | -0.596 | 0.551 | 0.625 |
| STG self | 4.06 | 1.473 | 2.757 | 0.006 | 57.963 |
| TPC other | 1.307 | 1.043 | 1.254 | 0.210 | 3.695 |
| Cerv.N13 self-other | -0.919 | 0.511 | -1.8 | 0.072 | 0.399 |
| HEP Intero | 0.056 | 0.033 | 1.7 | 0.089 | 1.058 |

| **Table S7: Descriptive Statistics of HEP AUC in the different diagnosis groups** | | | | | | | | | | | | | |
| --- | --- | --- | --- | --- | --- | --- | --- | --- | --- | --- | --- | --- | --- |
|  | | **schizophrenia** | | **schizoaffective** | | **non-specific** | | **delusional** | | **acute** | | **paranoid** | |
| Valid |  | 11 |  | 8 |  | 6 |  | 3 |  | 1 |  | 1 |  |
| Missing |  | 3 |  | 1 |  | 1 |  | 0 |  | 0 |  | 0 |  |
| Mean |  | -9.964 |  | -7.549 |  | -20.658 |  | -19.520 |  | -24.134 |  | -15.928 |  |
| Std. Deviation |  | 4.921 |  | 5.449 |  | 12.831 |  | 8.210 |  |  |  |  |  |
| Minimum |  | -19.063 |  | -15.250 |  | -38.712 |  | -27.158 |  | -24.134 |  | -15.928 |  |
| Maximum |  | -1.635 |  | 0.000 |  | -6.090 |  | -10.837 |  | -24.134 |  | -15.928 |  |
|  | | | | | | | | | | | | | |

**Figure S1: Flow-chart of participant attrition.** HBD = heartbeat detection task, SEP = somatosensory evoked potentials, TT = touch thresholds, fMRI = functional magnetic resonance imaging
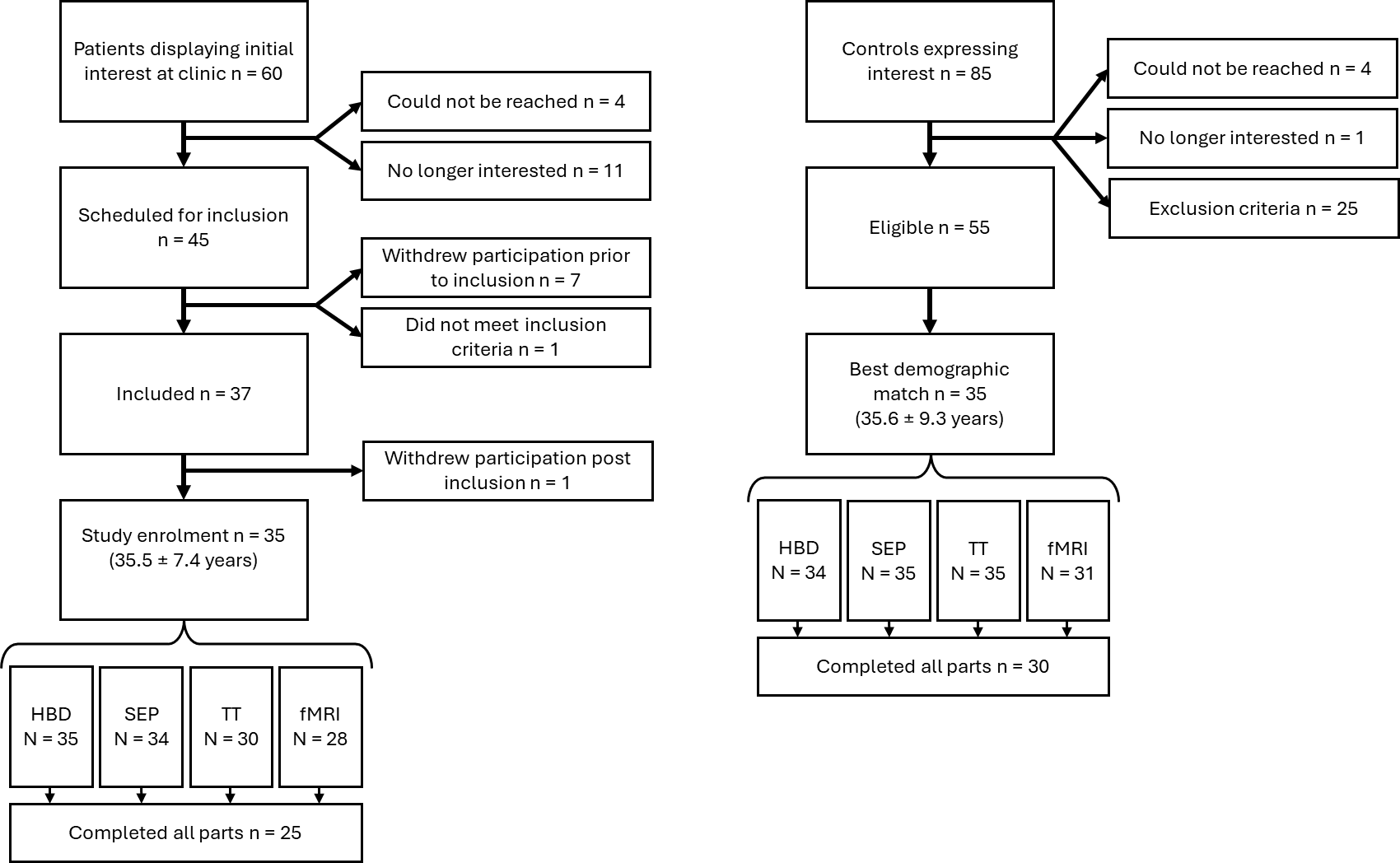


**Figure S2: Patients show increased brain activity compared to the controls across touch conditions (main effect of group).** Thresholded at p<0.001 for display purpose, color-bar indicates t-values.


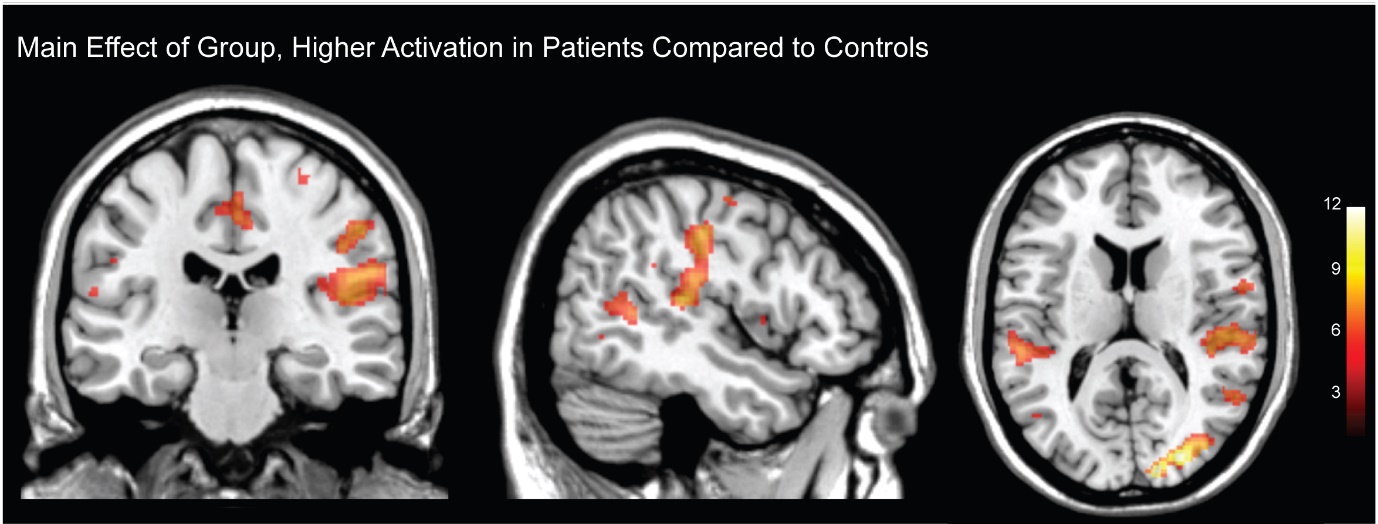


**References**

1. Salamone PC, Sedeño L, Legaz A, et al. Dynamic neurocognitive changes in interoception after heart transplant. *Brain Communications*. 2020;2(2)doi:10.1093/braincomms/fcaa095

2. Boehme R, Hauser S, Gerling GJ, Heilig M, Olausson H. Distinction of self-produced touch and social touch at cortical and spinal cord levels. *PNAS Proceedings of the National Academy of Sciences* 2019;

3. Boehme R, Karlsson MF, Heilig M, Olausson H, Capusan AJ. Sharpened self-other distinction in attention deficit hyperactivity disorder. *NeuroImage: Clinical*. 2020;27:102317.

4. Frost-Karlsson M, Capusan AJ, Perini I, et al. Neural processing of self-touch and other-touch in anorexia nervosa and autism spectrum condition. *NeuroImage: Clinical*. 2022;36:103264.

5. Leucht S, Samara M, Heres S, Davis JM. Dose equivalents for antipsychotic drugs: the DDD method. *Schizophrenia bulletin*. 2016;42(suppl_1):S90-S94.

6. Adviser S. What Are Dose Conversions from Oral to Injectable for the Long-Acting Injectable (LAI) Antipsychotic Medications Available in the U.S.? *American Psychiatric Association*. April 24 2024;

7. Hayasaka Y, Purgato M, Magni LR, et al. Dose equivalents of antidepressants: evidence-based recommendations from randomized controlled trials. *Journal of affective disorders*. 2015;180:179-184.

8. Salamone PC, Legaz A, Sedeño L, et al. Interoception Primes Emotional Processing: Multimodal Evidence from Neurodegeneration. *J Neurosci*. May 12 2021;41(19):4276-4292. doi:10.1523/jneurosci.2578-20.2021

9. Perakakis P. HEPLAB: a Matlab graphical interface for the preprocessing of the heartbeat-evoked potential. *Zenodo*. 2019;

10. Pollatos O, Schandry R. Accuracy of heartbeat perception is reflected in the amplitude of the heartbeat-evoked brain potential. *Psychophysiology*. 2004;41(3):476-482. doi:<https://doi.org/10.1111/1469-8986.2004.00170.x>

11. Gray MA, Taggart P, Sutton PM, et al. A cortical potential reflecting cardiac function. *Proceedings of the National Academy of Sciences*. 2007;104(16):6818-6823.

12. Tarvainen MP, Lipponen JA, Kuoppa P. Analysis and preprocessing of hrv—kubios HRV software. *ECG Time Series Variability Analysis*. CRC Press; 2017:159-186.

13. Kilteni K, Engeler P, Ehrsson HH. Efference Copy Is Necessary for the Attenuation of Self-Generated Touch. *iScience*. Feb 21 2020;23(2):100843. doi:10.1016/j.isci.2020.100843

14. Burin D, Pyasik M, Ronga I, Cavallo M, Salatino A, Pia L. “As long as that is my hand, that willed action is mine”: Timing of agency triggered by body ownership. *Consciousness and Cognition*. 2018/02/01/ 2018;58:186-192. doi:<https://doi.org/10.1016/j.concog.2017.12.005>

15. Haggard P. Sense of agency in the human brain. *Nature Reviews Neuroscience*. 2017/04/01 2017;18(4):196-207. doi:10.1038/nrn.2017.14

16. Hur J-W, Kwon JS, Lee TY, Park S. The crisis of minimal self-awareness in schizophrenia: A meta-analytic review. *Schizophrenia Research*. 2014/01/01/ 2014;152(1):58-64. doi:<https://doi.org/10.1016/j.schres.2013.08.042>

17. Maeda T, Takahata K, Muramatsu T, et al. Reduced sense of agency in chronic schizophrenia with predominant negative symptoms. *Psychiatry Res*. Oct 30 2013;209(3):386-92. doi:10.1016/j.psychres.2013.04.017

18. Blakemore SJ, Smith J, Steel R, Johnstone CE, Frith CD. The perception of self-produced sensory stimuli in patients with auditory hallucinations and passivity experiences: evidence for a breakdown in self-monitoring. *Psychol Med*. Sep 2000;30(5):1131-9. doi:10.1017/s0033291799002676

19. Kaldewaij R, Salamone P, Enmalm A, et al. Ketamine reduces the neural distinction between self-and other-produced affective touch-a double-blind placebo-controlled study. 2023;

20. Corlett PR, Honey GD, Fletcher PC. Prediction error, ketamine and psychosis: An updated model. *J Psychopharmacol*. Nov 2016;30(11):1145-1155. doi:10.1177/0269881116650087

21. Corlett PR, Honey GD, Fletcher PC. From prediction error to psychosis: ketamine as a pharmacological model of delusions. *J Psychopharmacol*. May 2007;21(3):238-52. doi:10.1177/0269881107077716

22. Keizer A, Heijman JO, Dijkerman HC. Do transdiagnostic factors influence affective touch perception in psychiatric populations? *Current Opinion in Behavioral Sciences*. 2022/02/01/ 2022;43:125-130. doi:<https://doi.org/10.1016/j.cobeha.2021.09.006>

23. Sailer U, Ackerley R. Exposure shapes the perception of affective touch. *Developmental Cognitive Neuroscience*. 2019/02/01/ 2019;35:109-114. doi:<https://doi.org/10.1016/j.dcn.2017.07.008>

24. Fraile, M., Salamone, P., Zoltowski, A., Quackenbush, W., Keceli-Kaysili, B., & Cascio, C. J. (2025). HEP_Preprocessing: EEG Preprocessing Pipeline for Heartbeat-Evoked Potentials (HEP) Analysis. [https://github.com/casciolab/HEP_Preprocessing](https://eur01.safelinks.protection.outlook.com/?url=https%3A%2F%2Fgithub.com%2Fcasciolab%2FHEP_Preprocessing&data=05%7C02%7Crebecca.bohme%40liu.se%7C5a600b261035424b1b1108dda990bc42%7C913f18ec7f264c5fa816784fe9a58edd%7C0%7C0%7C638853161270417318%7CUnknown%7CTWFpbGZsb3d8eyJFbXB0eU1hcGkiOnRydWUsIlYiOiIwLjAuMDAwMCIsIlAiOiJXaW4zMiIsIkFOIjoiTWFpbCIsIldUIjoyfQ%3D%3D%7C0%7C%7C%7C&sdata=Xm4hLTcv3Zy2%2BTPnUt4CWG9QqicpUHsi2IixgKk7GLU%3D&reserved=0)

25. de la Fuente, A., Sedeño, L., Vignaga, S. S., Ellmann, C., Sonzogni, S., Belluscio, L., García-Cordero, I., Castagnaro, E., Boano, M., Cetkovich, M., Torralva, T., Cánepa, E. T., Tagliazucchi, E., Garcia, A. M., & Ibañez, A. (2019). Multimodal neurocognitive markers of interoceptive tuning in smoked cocaine. Neuropsychopharmacology : official publication of the American College of Neuropsychopharmacology, 44(8), 1425–1434. [https://doi.org/10.1038/s41386-019-0370-3](https://eur01.safelinks.protection.outlook.com/?url=https%3A%2F%2Fdoi.org%2F10.1038%2Fs41386-019-0370-3&data=05%7C02%7Crebecca.bohme%40liu.se%7C5a600b261035424b1b1108dda990bc42%7C913f18ec7f264c5fa816784fe9a58edd%7C0%7C0%7C638853161270365692%7CUnknown%7CTWFpbGZsb3d8eyJFbXB0eU1hcGkiOnRydWUsIlYiOiIwLjAuMDAwMCIsIlAiOiJXaW4zMiIsIkFOIjoiTWFpbCIsIldUIjoyfQ%3D%3D%7C0%7C%7C%7C&sdata=JXsMjNyZBFbiIXbcbjFcMhrd7q6zeyFW1WZsfx4fztI%3D&reserved=0)

26. Fittipaldi, S., Abrevaya, S., Fuente, A., Pascariello, G. O., Hesse, E., Birba, A., Salamone, P., Hildebrandt, M., Martí, S. A., Pautassi, R. M., Huepe, D., Martorell, M. M., Yoris, A., Roca, M., García, A. M., Sedeño, L., & Ibáñez, A. (2020). A multidimensional and multi-feature framework for cardiac interoception. NeuroImage, 212, 116677. [https://doi.org/10.1016/j.neuroimage.2020.116677](https://eur01.safelinks.protection.outlook.com/?url=https%3A%2F%2Fdoi.org%2F10.1016%2Fj.neuroimage.2020.116677&data=05%7C02%7Crebecca.bohme%40liu.se%7C5a600b261035424b1b1108dda990bc42%7C913f18ec7f264c5fa816784fe9a58edd%7C0%7C0%7C638853161270389934%7CUnknown%7CTWFpbGZsb3d8eyJFbXB0eU1hcGkiOnRydWUsIlYiOiIwLjAuMDAwMCIsIlAiOiJXaW4zMiIsIkFOIjoiTWFpbCIsIldUIjoyfQ%3D%3D%7C0%7C%7C%7C&sdata=IIudBZ4bSVmRu%2Blq4x4MlDqaFYloRG1Zze7L2wufvlg%3D&reserved=0)

27. Fraile Vazquez, M. E., Cascio, C., Salamone, P. C., Hazelton, J., Emina, F., & Prinsen, J. (2025, May 12). D’-tecting the Beat: Refining d’ in Heartbeat Detection Tasks. [https://doi.org/10.31234/osf.io/4unep_v4](https://eur01.safelinks.protection.outlook.com/?url=https%3A%2F%2Fdoi.org%2F10.31234%2Fosf.io%2F4unep_v4&data=05%7C02%7Crebecca.bohme%40liu.se%7C5a600b261035424b1b1108dda990bc42%7C913f18ec7f264c5fa816784fe9a58edd%7C0%7C0%7C638853161270403871%7CUnknown%7CTWFpbGZsb3d8eyJFbXB0eU1hcGkiOnRydWUsIlYiOiIwLjAuMDAwMCIsIlAiOiJXaW4zMiIsIkFOIjoiTWFpbCIsIldUIjoyfQ%3D%3D%7C0%7C%7C%7C&sdata=G8JO0K6%2B1rWcbsXGc5%2Bldf5irBnfxoiUloxlg0Twf5c%3D&reserved=0)
